# Epigenetic reprogramming of airway macrophages promotes polarization and inflammation in muco-obstructive lung disease

**DOI:** 10.1038/s41467-021-26777-9

**Published:** 2021-11-11

**Authors:** Joschka Hey, Michelle Paulsen, Reka Toth, Dieter Weichenhan, Simone Butz, Jolanthe Schatterny, Reinhard Liebers, Pavlo Lutsik, Christoph Plass, Marcus A. Mall

**Affiliations:** 1 Division of Cancer Epigenomics, German Cancer Research Center (DKFZ), Heidelberg, Germany; 2 Ruprecht Karl University of Heidelberg, Heidelberg, Germany; 3 Translational Lung Research Center Heidelberg (TLRC), German Center for Lung Research (DZL), Heidelberg, Germany; 4 Department of Translational Pulmonology, University of Heidelberg, Heidelberg, Germany; 5 Division of Molecular Thoracic Oncology, German Cancer Research Center (DKFZ), Heidelberg, Germany; 6 Department of Pediatric Respiratory Medicine, Immunology and Critical Care Medicine, Charité-Universitätsmedizin Berlin, Corporate member of Freie Universität Berlin and Humboldt-Universität zu Berlin, Berlin, Germany; 7 Berlin Institute of Health at Charité – Universitätsmedizin Berlin, Berlin, Germany; 8 German Center for Lung Research (DZL), Associated Partner, Berlin, Germany; 9 Present Address: Novo Nordisk Foundation Center for Stem Cell Biology, University of Copenhagen, Copenhagen, Denmark; 10 Present Address: National Center for Tumor Diseases (NCT), Heidelberg, Germany

**Keywords:** Epigenetics, Alveolar macrophages, Chronic obstructive pulmonary disease, Cystic fibrosis

## Abstract

Lung diseases, such as cystic fibrosis and COPD, are characterized by mucus obstruction and chronic airway inflammation, but their mechanistic link remains poorly understood. Here, we focus on the function of the mucostatic airway microenvironment on epigenetic reprogramming of airway macrophages (AM) and resulting transcriptomic and phenotypical changes. Using a mouse model of muco-obstructive lung disease (*Scnn1b*-transgenic), we identify epigenetically controlled, differentially regulated pathways and transcription factors involved in inflammatory responses and macrophage polarization. Functionally, AMs from *Scnn1b*-transgenic mice have reduced efferocytosis and phagocytosis, and excessive inflammatory responses upon lipopolysaccharide challenge, mediated through enhanced *Irf1* function and expression. Ex vivo stimulation of wild-type AMs with native mucus impairs efferocytosis and phagocytosis capacities. In addition, mucus induces gene expression changes, comparable with those observed in AMs from *Scnn1b*-transgenic mice. Our data show that mucostasis induces epigenetic reprogramming of AMs, leading to changes favoring tissue damage and disease progression. Targeting these altered AMs may support therapeutic approaches in patients with muco-obstructive lung diseases.

## Abstract (web summary)

Muco-obstructive lung diseases are characterised by airway macrophage (AM) populations which may have epigenetic changes. Here using a mouse model the authors show epigenetic alteration of AMs with changes in LPS response, phagocytosis and efferocytosis similar to culture with mucus in vitro.

## Introduction

Mucus obstruction and chronic airway inflammation are important features of a spectrum of muco-obstructive lung diseases. Amongst those are rare mono-genetic disorders such as cystic fibrosis (CF) and primary ciliary dyskinesia, and common complex lung diseases such as chronic obstructive pulmonary disease (COPD) and asthma^1–3^. Emerging evidence suggests that excess mucus per se can trigger chronic airway inflammation, as shown in *Scnn1b-*Tg mice that share key features with CF and COPD, such as mucus plugging, chronic airway inflammation, and emphysema-like structural lung damage, even in the absence of bacterial infection^4–10^.

Airway macrophages (AM) reside in the lumen of the conducting airways and distal airspaces and are important for lung homeostasis and host defense. AMs show a high degree of plasticity, maintaining adequate immunological responses against invading pathogens while avoiding pro-inflammatory responses to debris or inhaled particles. This led to the simplified classification of macrophages into proinflammatory M1 and anti-inflammatory M2 polarization states^11^. In muco-obstructive lung diseases, AMs are activated, dysfunctional and often correlate with disease pathogenesis and severity^12^. Aberrant changes include impairments in efferocytosis, bacterial clearance and lysosomal killing, survival, and increased release of inflammatory mediators^13–18^. Depleting AMs in mice with muco-obstructive lung disease leads to increased airway inflammation, an increase in mucus cells and mucus plugging, as well as an increase in lethal pneumonia, highlighting the critical function of macrophages in promoting lung pathology in muco-obstructive disease^19,20^.

Previous studies point to epigenetic regulation of the polarization of macrophages, with the lung microenvironment being important in shaping the distinct transcriptional and epigenetic landscape of resident AMs and thereby modulating their differentiation, plasticity, cellular identity, and functions^21–24^. This was shown by replacing tissue-resident lung macrophages with bone marrow-derived macrophages, which acquired a lung-specific gene expression profile and restored functional properties similar to the original lung macrophages^25^. Even the transfer of fully differentiated peritoneal macrophages into the lung led to the upregulation of genes specific for AMs, highlighting the potential of the microenvironment to shape and reprogram macrophage identity independent of their development^23^. Excess mucus production and plugging, as common features of the microenvironment of chronic obstructive lung diseases, could potentially drive dysregulated responses of AMs through epigenetic priming and thereby support disease progression^12^. However, these interactions have not been addressed to date.

We hypothesize that airway mucus obstruction induces epigenetic changes in AMs, which alter their phenotype and functional responses. To test this hypothesis, we elucidate genome-wide epigenetic and transcriptional changes in AMs from *Scnn1b-*Tg and wild-type (WT) mice. Additionally, high dimensional flow cytometry is utilized to assess AM activation and heterogeneity on a single-cell level. The functional consequences of mucus obstruction on AM phagocytosis and efferocytosis are determined by flow cytometry. Further, we evaluate the response to bacterial-derived lipopolysaccharides (LPS) on cytokine expression and perform an integrated analysis of assay for transposase accessible chromatin sequencing (ATACseq) and RNA sequencing (RNAseq) to identify differentially active transcription factors involved in the transcriptional induction of inflammatory programs in AMs. Finally, the effects of native mucus on AM activation and plasticity are evaluated on gene and protein expression levels, and functional changes are assessed via flow cytometry. This showed that muco-obstruction and mucus exposure is associated with a mixed AM phenotype and pathophysiologically relevant changes in AM immune responses and functions.

## Results

### AMs from muco-obstructive mice are epigenetically distinct from WT AMs

**Fig. 1:**
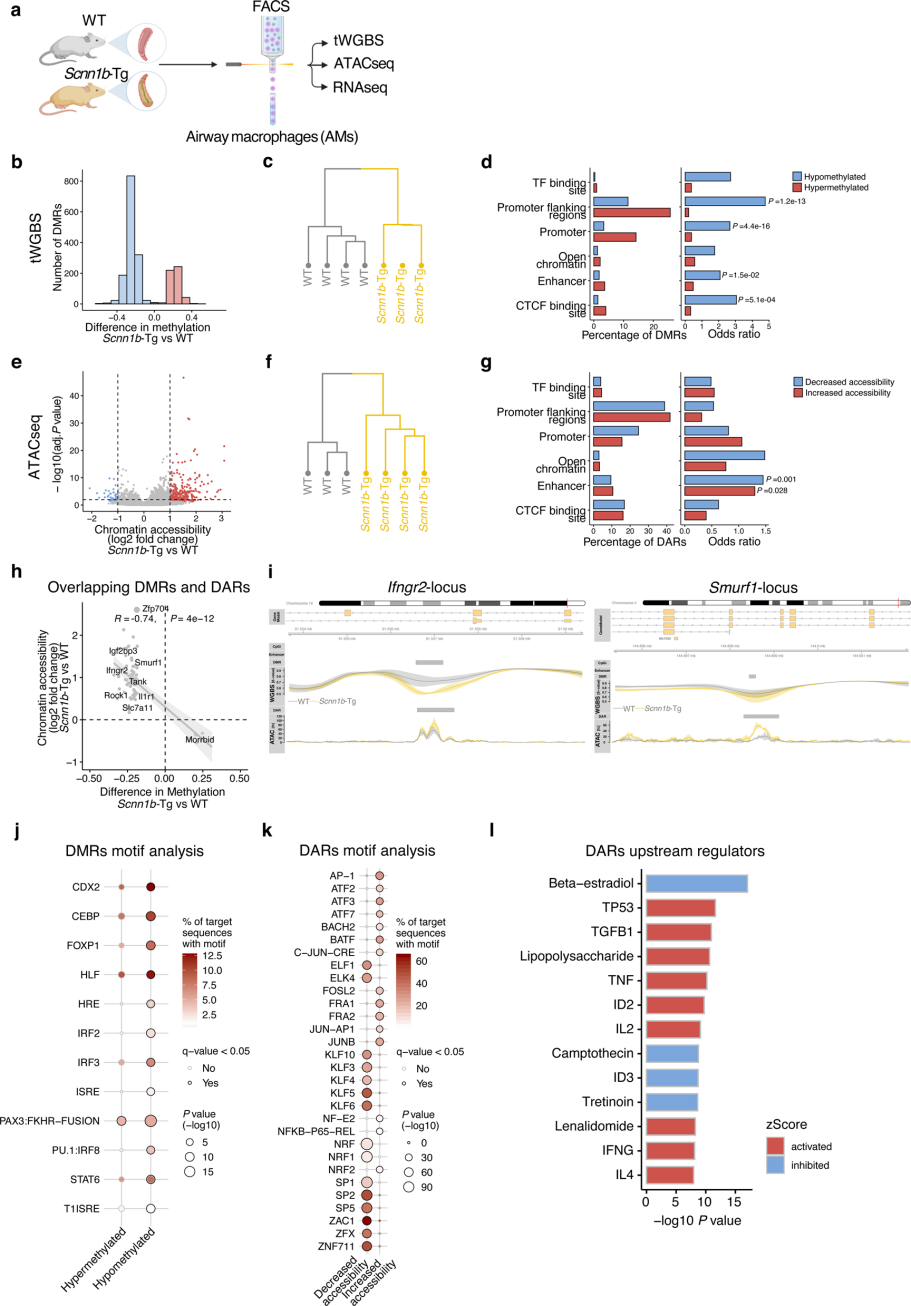
AMs from mice with muco-obstructive lung disease are epigenetically distinct from WT AMs. **a** Graphical representation of the experimental workflow, created with www.biorender.com. **b** Distribution of *Scnn1b*-transgenic (Tg) vs. wild-type (WT) airway macrophage (AM) DNA methylation differences of differentially methylated regions (DMR), defined by DSS. DMRs are characterized by at least three CpGs with an adjusted (adj.) *P* value < 0.05, a width of >50 bp, and an average change of methylation >0.1. Hierarchical clustering of **c** DMRs methylation levels and **f** differentially accessible regions (DAR) accessibility. **e** Volcano plot of the chromatin accessibility analysis, performed with DiffBind. DARs: adj. *P* value < 0.05, absolute log2 fold change >1. Red dots: increased accessibility in *Scnn1b*-Tg AMs; blue dots reduced accessibility in *Scnn1b*-Tg AMs. Annotation and enrichment of **d** DMRs and **g** DARs to gene regulatory regions, as determined by the LOLA method. **h** Integrated analysis of overlapping DARs and DMRs. The gray diagonal represents the linear regression. Shaded areas are confidence intervals of the correlation coefficient at 95%. Correlation coefficients and *P* values were calculated by the Pearson correlation method. **i** Locus plot of selected DMRs and DARs showing average methylation and chromatin accessibility of *Scnn1b*-Tg AMs and WT AMs. Shaded areas indicate 95% confidence intervals. Motif enrichment of **j** DMRs and **k** DARs (adj. *P* value < 0.05) stratified in hypo- and hypermethylated DMRs and open and closed DARs, respectively. **l** Predicted upstream regulator analysis of chromatin accessibility changes. Tagmentation-based whole-genome bisulfite sequencing (tWGBS), *Scnn1b-*Tg (*n* = 3) vs. WT (*n* = 4); Assay for transposase-accessible chromatin sequencing (ATACseq), *Scnn1b-*Tg (*n* = 4) vs. WT (*n* = 3). Source data are provided as a Source Data file.

To understand the role of the airway microenvironment in muco-obstructive lung disease on the epigenetic landscape of AMs, we isolated resident AMs to investigate DNA methylation (tWGBS, tagmentation-based whole-genome bisulfite sequencing), chromatin accessibility (ATACseq), and gene expression (RNAseq). We isolated AMs (CD45^+^Siglec-F^+^CD11c^+^) from *Scnn1b*-Tg mice and WT controls by fluorescence-activated cell sorting (FACS) with a purity of >95% (Fig. 1a and Supplementary Fig. 1a, b). To verify that transgenic overexpression of *Scnn1b* under control of the club cell secretory protein (CCSP) promoter is not present in AMs, we stained AMs from *Scnn1b*-Tg and WT mice with fluorescently-labeled antibodies against SCNN1B and the macrophage-specific receptor MerTK. Fluorescence microscopic analysis confirmed the absence of SCNN1B in MerTK^+^ AMs from *Scnn1b*-Tg as well as WT mice. In comparison, a strong signal for SCNN1B was detectable in cultured mouse tracheal epithelial cells (mTEC) (Supplementary Fig. 1c). Consequently, changes in the epigenome and transcriptome can be attributed to an altered airway microenvironment in muco-obstructive lung disease.

Airway macrophages are long-lived and self-maintained with minor contributions of the circulating bone marrow-derived monocyte pool^26^. However, monocytes enter the lung under inflammatory conditions and potentially convert into AMs, eventually resembling the resident population^27^. To evaluate the role of monocyte-to-AM transition in the *Scnn1b*-Tg mouse model, we applied a computational deconvolution method^28^ to compare the cell type composition in our RNAseq data with single-cell RNAseq data characterizing monocyte and macrophage populations in homeostasis and inflammation of the lung^29,30^. Deconvolution revealed a homogenous composition of resident AMs in all *Scnn1b*-Tg and WT samples (Supplementary Fig. 1d, e). Additionally, the comparison with gene expression changes observed in resident vs. recruited AMs, identified in a lineage-tracing model of lung fibrosis^27^, depicted no correlation (Pearson correlation: *R* = 0.03) with the gene expression changes observed in our RNAseq data (Supplementary Fig. 1f). Overall, these results imply that the sorted AM populations in our study are homogeneous, and monocyte-to AM transition can be neglected in the *Scnn1b-*Tg mouse model of muco-obstruction.

Global DNA-methylation analysis with single CpG resolution in WT and *Scnn1b*-Tg AMs displayed a similar, bimodal *β*-value distribution, as expected for homogenous cell populations (Supplementary Fig. 2a). No significant changes in global DNA methylation (average CpG ß-value = 0.73) levels were identified between the two groups. Differential methylation analysis, comparing *Scnn1b*-Tg and WT AMs, revealed 1926 differentially methylated regions (DMRs; Fig. 1b and Supplementary Data 1) associated with 1625 different genes. The majority of DMRs (1404) showed reduced methylation in *Scnn1b*-Tg AMs. Comparison of the chromatin accessibility at identified ATACseq peaks revealed 390 differentially accessible regions (DARs; Fig. 1e and Supplementary Data 2), with the majority having increased accessibility in *Scnn1b*-Tg AMs (339 DARs). The 390 DARs were associated with 312 different genes. As expected, hierarchical clustering of DMRs and DARs separated AMs according to their genotypes (Fig. 1c, f), confirming that distinct changes are present in AMs from *Scnn1b*-Tg mice. Annotation and enrichment analysis of DMRs and DARs, found hypomethylated DMRs enriched in promoters, promoter flanking regions, enhancers, and CTCF-binding sites (Fig. 1d). DARs showed a similar pattern, with enrichment in enhancer regions (Fig. 1g). A significant inverse correlation between DMRs and DARs further supported the association between decreased DNA methylation and open chromatin and vice versa (Fig. 1h). We identified an overlap between DMRs and DARs, with a strong and significant inverse correlation (Pearson correlation: *R* = −0.74; *P* value < 0.001), associated with 58 genes. This included coherently hypomethylated loci with increased accessibility in *Scnn1b*-Tg AMs for genes involved in cytokine signaling, such as *Tank*, *Il1r1*, or the negative regulator *Smurf1*, as well as genes previously shown to be associated with chronic lung diseases (e.g., *Infgr2*, *Zfp704*, and *Sec14l1*) (Fig. 1i and Supplementary Fig. 2b)^31–33^. To identify transcription factor (TF) binding sites that potentially mediate gene regulatory changes upon alterations in DNA-methylation or chromatin accessibility, we performed TF motif analysis on DMRs and DARs. Hypomethylated DMRs and DARs in *Scnn1b*-Tg AMs were significantly enriched for TFs known to regulate inflammatory responses and macrophage polarization. For example, we identified highly enriched motifs for C/EBP, IRF2, IRF3, and STAT6 (Fig. 1j) in DMRs and motifs for the inflammation associated TFs JUNB, FRA1, NFkB-p65 (RELA), and the M2 polarizing TF ATF3 in DARs (Fig. 1k)^34–38^. Prediction of potential master regulators of changes in chromatin accessibility revealed cytokines such as TGF-β, TNF, IFN-γ, IL-4, as well as bacterial-derived LPS as activators in *Scnn1b*-Tg AMs (Fig. 1l). These predicted master regulators support the roles of the aforementioned TFs involved in their signaling pathways^39^. The results indicate a role of the muco-obstructive airway microenvironment in modulating the epigenetic makeup of AMs in regions that potentially allow the binding of TFs associated with inflammatory processes, macrophage polarization, and activation.

### Transcriptional activation of AMs coincides with epigenetic changes

**Fig. 2:**
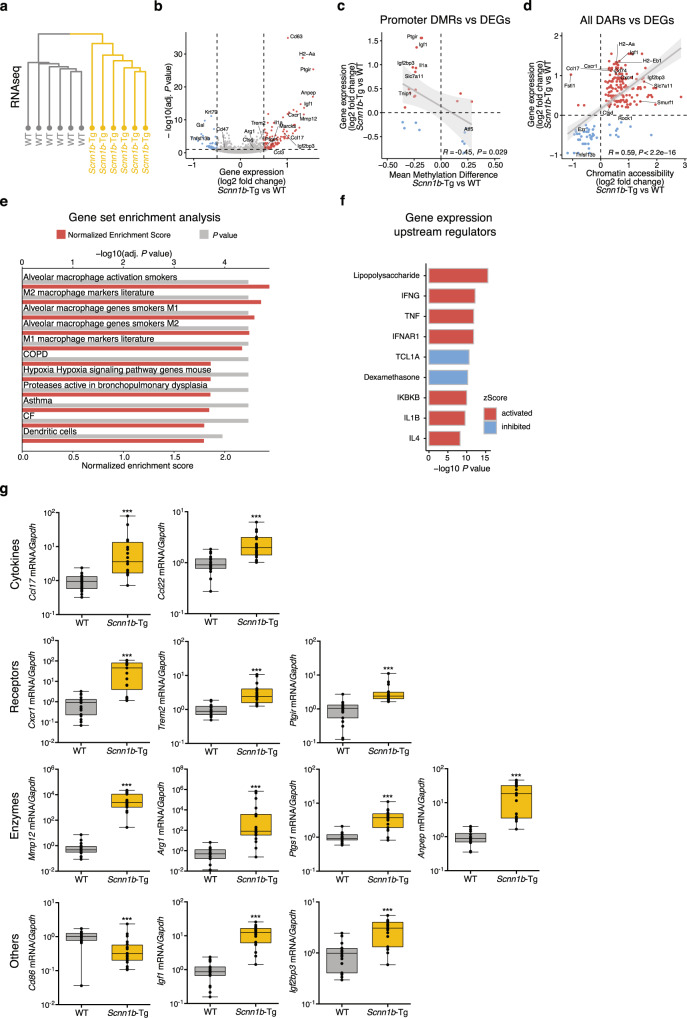
Transcriptional activation of *Scnn1b*-Tg AMs coincides with epigenetic patterns of reduced methylation and increased chromatin accessibility. **a** Unsupervised hierarchical clustering of scaled gene expression in *Scnn1b*-transgenic (Tg) and wild-type (WT) airway macrophages (AM). **b** Volcano plot of differential gene expression analysis. Differentially expressed genes (DEG), are defined by DESeq2: adjusted (adj.) *P* value < 0.1, absolute log2 fold change >0.5. Red dots: increased expression in *Scnn1b*-Tg AMs; blue dots reduced expression in *Scnn1b*-Tg AMs. **c** Integrated analysis of gene expression and promoter DNA methylation changes. Methylation differences of promoter differentially methylated regions (DMR) (<5 kbs from transcriptional start sites) vs. expression changes of the corresponding DEGs (adj. *P* value < 0.1). The gray diagonal represents the linear regression. Shaded areas are confidence intervals of the correlation coefficient at 95%. Correlation coefficients and *P* values were calculated by the Pearson correlation method. **d** Integrated analysis of gene expression and chromatin accessibility changes. Log2 fold change of differentially accessible regions (DARs) (adj. *P* value < 0.05) and DEGs (adj. *P* value < 0.1). The gray diagonal represents the linear regression. Shaded areas are the confidence intervals of the correlation coefficient at 95%. Correlation coefficients and *P* values were calculated by the Pearson correlation method. **e** Gene set enrichment analysis using custom gene sets relevant to lung diseases and cellular physiology^47^. **f** Predicted upstream regulator analysis of gene expression changes. **g** Gene expression for *Ccl17, Ccl22, Cxcr1, Trem2, Ptgir, Mmp12, Arg1, Ptgs1, Anpep, Cd86, Igf1*, and *Igf2bp3* was measured by qPCR. Box plots indicate the largest value within the 1.5 times interquartile range above 75th percentile, 75th percentile, median, 25th percentile, and smallest value within the 1.5 times interquartile range below 25th percentile of **g** *n* = 20 per group (*Ccl17, Ccl22, Trem2*), *n* = 19 per group (*Ptgir*, *Mmp12, Arg1, Ptgs1, Anpep, Igf1, Igf2bp3)*, *n* = 18 WT, *n* = 17 *Scnn1b*-Tg (*Cxcr1*), *n* = 19 WT, *n* = 20 *Scnn1b*-Tg (*Cd86)*. *P value < 0.05; **P value < 0.01*;* ***P value < 0.001 by Mann–Whitney *U*-test. RNA sequencing (RNAseq), *n* = 6 per group. Source data are provided as a Source Data file.

DNA methylation and chromatin accessibility are two key regulators of transcription. Similar to the results obtained from the DNA methylome and chromatin accessibility analysis, gene expression profiles showed substantial differences between AMs from *Scnn1b*-Tg and WT mice. Hierarchical clustering identified two groups representing *Scnn1b*-Tg AMs and WT AMs (Fig. 2a). This suggests that gene expression, DNA methylation, and chromatin accessibility patterns are interconnected. Indeed, transcriptional activation correlated with promoter hypomethylation and open chromatin, and transcriptional inhibition with DNA hypermethylation and closed chromatin, respectively (Pearson correlation: Promoter DMRs vs. DEGs: *R* = −0.45, *P* value < 0.05; DARs vs. DEGs: *R* = 0.59, *P* value < 0.001) (Fig. 2c, d). Prominent examples of promoter hypomethylation and increased gene expression in *Scnn1b*-Tg AMs were interleukin 1 alpha (*Il1a*) and insulin-like growth factor 1 (*Igf1*) (Supplementary Fig. 3c). *Il1a* is a cytokine previously associated with sterile inflammation and tissue damage in children with CF^6,40–42^. *Igf1* is a pleiotropic growth factor associated with lung development, tissue remodeling, and inflammation in various lung diseases such as CF, COPD, asthma, and acute lung injury^43^. Correlation analysis of chromatin accessibility and transcriptional regulation identified genes associated with macrophage polarization (*Ccl17*, *Cxcr1*, *H2-Aa*, *H2-Ab1*), inflammation (*Ezr*, *CD74*), and remodeling (*Ctsd*) (Fig. 2d). *Ezr* (ezrin) is important for Toll-like receptor (TLR) 4-mediated LPS signaling and bacterial host defense in macrophages^44^. The AM surface marker CD74 contributes to neutrophil accumulation in the airways^45^, and *Ctsd* (cathepsin D) is associated with emphysema in smoke models^46^. Overall, differential gene expression analysis revealed 117 significantly upregulated and 46 significantly downregulated genes in *Scnn1b*-Tg AMs compared to WT AMs (Fig. 2b and Supplementary Data 3). Enrichment of differentially expressed genes (DEGs) with pathways and gene ontologies revealed terms associated with migration (e.g., leukocyte migration and regulation of cell migration), immune processes (e.g., chemokine signaling pathway, neutrophil degranulation, and cytokine production), and tissue remodeling (Supplementary Fig. 3a)—all previously defined hallmarks of the *Scnn1b*-Tg mouse model and muco-obstructive lung diseases^1,4,6,15^. To obtain a more comprehensive picture of specific biological pathways involved in AM alterations, we utilized previously published gene sets relevant for lung diseases and cell physiology^47^. Gene set enrichment analysis (GSEA) revealed a strong involvement of genes associated with AM activation and plasticity, enabling adaptation to environmental perturbations in homeostasis. These genes are frequently deregulated in cancer^48^, atherosclerosis^49^, and rheumatoid arthritis^50^. Both M1 and M2 signature genes were markedly upregulated in *Scnn1b*-Tg AMs (Fig. 2e), and genes related to muco-obstructive lung diseases such as CF, COPD, and asthma, were positively enriched in *Scnn1b*-Tg AMs (Fig. 2e and Supplementary Fig. 3b). Differential expression of these markers and other cytokines, receptors, and enzymes was independently verified by qRT-PCR (Fig. 2g). Upstream regulator analysis identified potential activators involved in the induction of type I immunity, such as LPS, TNF, IFN-γ, but also inducers of type II immune responses, such as IL-4. Those activators overlapped with mediators identified in our ATACseq dataset (Fig. 1l). Additionally, IFNAR, IKBKB, and IL-1β were predicted upstream activators (Fig. 2f).

Together, these data show a strong association between the microenvironmentally driven epigenetic changes observed in DNA methylation and chromatin accessibility with transcriptional activation of genes implicated in immune processes and tissue remodeling in AMs of *Scnn1b*-Tg mice.

### Flow cytometry validates AM activation and indicates a mixed phenotype

**Fig. 3:**
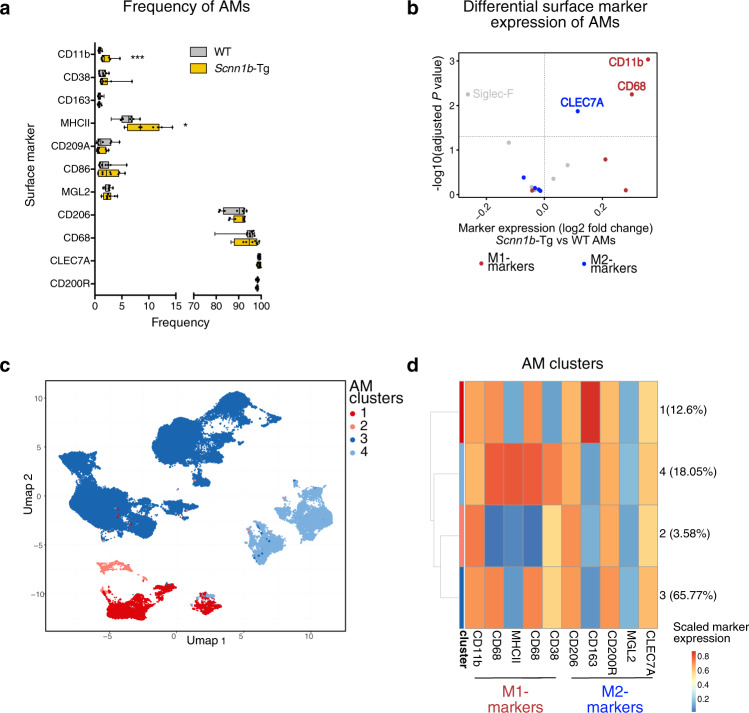
Single-cell analysis of macrophage surface marker expression validates enhanced activation of *Scnn1b*-Tg AMs. **a** Frequency of indicated surface markers expressed by airway macrophages (AM). **b** Differential surface marker expression of *Scnn1b*-transgenic (Tg) vs. wild-type (WT) AMs, defined by cluster analysis. Red dots: surface markers for classical macrophage polarization (M1); blue dots: surface markers for alternative macrophage polarization (M2). **c** Uniform Manifold Approximation and Projection (UMAP) of 50,000 randomly sampled *Scnn1b*-Tg and WT AMs. **d** Scaled M1 and M2 surface marker expression of AM clusters. Box plots indicate the largest value within the 1.5× interquartile range above 75th percentile, 75th percentile, median, 25th percentile, and smallest value within the 1.5× interquartile range below 25th percentile of **a** *n* = 10 per group. *P value < 0.05; **P value < 0.01; ***P value < 0.001 by Mann–Whitney *U*-test. CD11b, *P* < 0.001; MHCII, *P* = 0.0185. Source data are provided as a Source Data file.

To validate macrophage activation on the protein level and assess AM heterogeneity, we performed single-cell high-dimensional flow cytometry (Supplementary Fig. 4a). We stained for surface markers associated with lung cell types (CD45.2, Siglec-F, CD11c, CD64, MerTK, and CD163) and macrophage activation and polarization (M1: CD11b, CD86, MHCII, CD68, CD38; M2: CD206, CD163, CD200R, CD209A, MGL2, CLEC7A). To further distinguish different populations of the innate and adaptive immune response, unsupervised clustering was performed. Cell types were assigned by cross-referencing cluster-specific marker genes with known cell type markers (Supplementary Fig. 4b) and were further visualized by uniform manifold approximation and projection (UMAP) (Supplementary Fig. 4c). Quantification of surface markers revealed an increased frequency of CD11b^+^ and MHCII^+^ AMs in *Scnn1b*-Tg lungs (Fig. 3a). Additionally, differential expression analysis of surface markers depicted a reduction in Siglec-F and an increase in CD11b, CLEC7A, and CD68 on *Scnn1b*-Tg compared to WT AMs (Fig. 3b), validating the enhanced activation of AMs in muco-obstructive lungs.

We further clustered WT and *Scnn1b*-Tg AMs into common sub-clusters to comprehensively evaluate AM heterogeneity. In total, four clusters were identified, present in *Scnn1b-*Tg and WT lungs (Fig. 3c). Notably, all clusters were represented by M1 and M2 activation markers (Fig. 3d). Cluster 4 was uniquely characterized by MHCII expression, and cluster 1 was represented by CD163 expressing AMs. According to the overall increase in MHCII^+^ AMs in *Scnn1b*-Tg lungs, an increase in the abundance of cluster 4 and a decrease in cluster 3 was observed in *Scnn1b*-Tg lungs (Supplementary Fig. 4d). Together, these results indicate a mixed phenotype of AMs, in contrast to an exclusive classification in pro-inflammatory (M1) or anti-inflammatory (M2) macrophage functions.

### AM-specific functions are impaired in *Scnn1b*-Tg mice

**Fig. 4:**
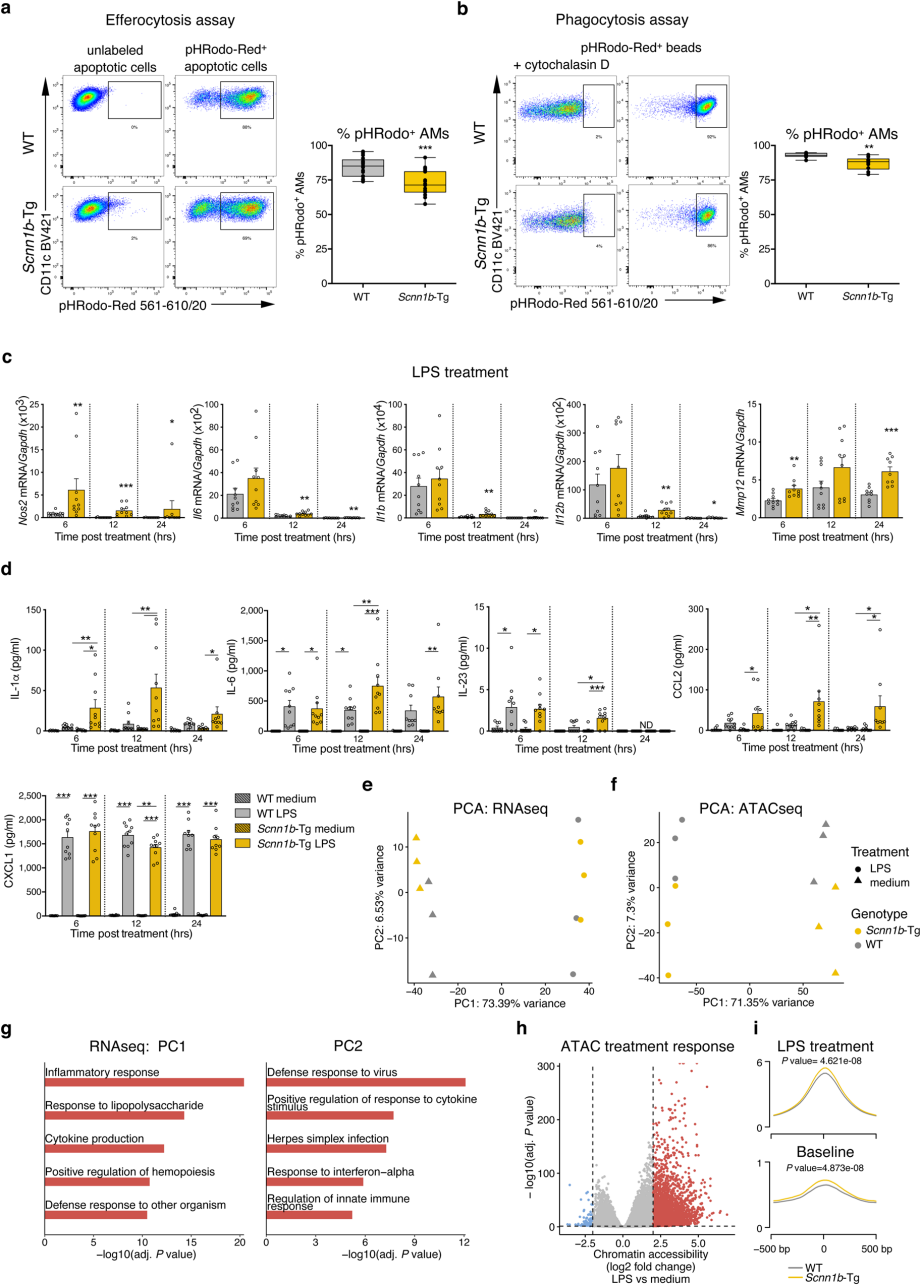
AM-specific functions are impaired in *Scnn1b*-Tg mice. Representative flow cytometry plots and percentage of pHRodo^+^ airway macrophages (AM), depicting **a** efferocytosis and **b** phagocytosis capacities. **c** Gene expression of *Nos2, Il6, Il1b, Il12b*, and *Mmp12*, assessed by qPCR and **d** protein expression of IL-1α, IL-6, IL-23p40/p19, CCL2, and CXCL1, assessed by cytokine bead array, at indicated time points post lipopolysaccharide (LPS) treatment. PCA of **e** RNA sequencing (RNAseq) and **f** Assay for transposase-accessible chromatin sequencing (ATACseq) data from *Scnn1b*-transgenic (Tg) vs. wild-type (WT) AMs treated with lipopolysaccharide (LPS) for 12 h (h). **g** Functional enrichment analysis of the top 100 genes explaining PC1 (left panel) and PC2 (right panel). **h** Volcano plot visualizing the treatment response (LPS vs. medium) on chromatin accessibility level. differentially accessible regions (DARs): adjusted (adj.) *P* value < 0.05; absolute log2 fold change >2. Red dots: increased accessibility in LPS treated AMs; blue dots reduced accessibility in LPS treated AMs. **i** Profile plot of all identified LPS responsive DARs, visualized for LPS treated *Scnn1-*Tg and WT AMs (top panel) and primary uncultured *Scnn1-*Tg and WT AMs at baseline (lower panel). Box plots indicate the largest value within the 1.5× interquartile range above 75th percentile, 75th percentile, median, 25th percentile, and smallest value within the 1.5× interquartile range below 25th percentile of **a** *n* = 17 WT, *n* = 19 *Scnn1b*-Tg, and **b** *n* = 10 per group. *P value < 0.05; **P value < 0.01; ***P value < 0.001 per group by Mann–Whitney *U*-test. **a** *P* < 0.001, **b** *P* = 0.0011. Bar plots show mean ± SEM of **c** *n* = 10 per group (6, 12 h) and *n* = 9 per group (24 h). *P value < 0.05; **P value < 0.01; ***P value < 0.001 per group by Mann–Whitney *U*-test. *Nos2*: 6 h *P* = 0.0021, 12 h *P* < 0.001, 24 h *P* = 0.178; *Il6*: 12 h *P* = 0.0052, 24 h *P* = 0.0078; *Il1b*: 12 h *P* = 0.0048; *Il12b*: 12 h *P* = 0.0089, 24 h *P* = 0.0314; *Mmp12*: 6 h *P* = 0.0021, 12 h *P* = 0.0887, 24 h *P* < 0.001. **d** Lipopolysaccharide (LPS) treatment: IL-1α, IL-23p40/p19, IL-6, CXCL1: *n* = 10 per group (6, 12 h), *n* = 9 per group (24 h); CCL2: *n* = 10 wt (6, 12 h), *n* = 9 wt (24 h), *n* = 9 *Scnn1b*-Tg (6, 12, 24 h). Medium control: IL-1α, IL-23p40/19, CCL2, CXCL1: n = 8 WT (6 h), *n* = 10 WT (12 h), *n* = 9 WT (24 h), *n* = 9 *Scnn1b*-Tg (6, 12, 24 h), IL-6: *n* = 9 WT (6 h), *n* = 10 WT (12 h), n = 9 WT (24 h), *n* = 9 *Scnn1b*-Tg (6, 12, 24 h). *P value < 0.05; **P value < 0.01; ***P value < 0.001 by One-Way ANOVA followed by Tukey´s post hoc test. **e**–**i** *n* = 3 per group. ND not detectable. Source data are provided as a Source Data file.

In chronic airway inflammation, the clearance of apoptotic cells by efferocytosis is central to prevent secondary necrosis and the release of danger-associated molecular patterns from dying cells^51^. We, therefore, analyzed the efferocytosis capacities of *Scnn1b*-Tg AMs and WT AMs in vivo by intratracheal instillation of apoptotic cells labeled with a pH-sensitive dye (pHRodo), which emits fluorescence under acidic conditions as found in the lysosome of AMs. Flow cytometry analysis revealed that fewer *Scnn1b*-Tg AMs stained positive for pHRodo, reflecting a reduced capability in the clearance of apoptotic cells (Fig. 4a and Supplementary Fig. 7a).

Another major problem in muco-obstructive lung diseases is bacterial airway infections, triggering acute exacerbations and disease progression^52^. We evaluated the phagocytic capacities of AMs, using pHRodo labeled *E. coli* particles and identified a significant reduction of phagocytosis in *Scnn1b*-Tg AMs compared to WT AMs (Fig. 4b and Supplementary Fig. 7c). To assess the role and responses of AMs upon bacterial stimulation, we treated primary AMs from *Scnn1b*-Tg and WT mice with bacterial LPS for 6, 12, and 24 h. The baseline expression for the pro-inflammatory cytokines *Il1b*, *Il6*, *Il12b*, and *Tnf* was not significantly changed in *Scnn1b*-Tg vs. WT AMs (Supplementary Fig. 5a). Treatment with LPS resulted in a strong inflammatory response from *Scnn1b*-Tg and WT AMs on RNA and protein level, with comparable immune responses for *Nos2*, *Il6*, *Il1b*, and *Il12b* (Fig. 4c) on mRNA and for IL-6 and IL-23 (Fig. 4d) on protein level. Protein levels for IL-1α and CCL2 were already significantly upregulated in *Scnn1b*-Tg AMs at 6 h. Protein levels for IL-1β and IL-12 were below the detection limit, and TNF expression was induced to similar levels in both genotypes. LPS responses are tightly regulated and self-limiting to reduce inflammation and tissue damage^53^. Accordingly, we observed reduced expression for most of the analyzed genes after 12 h of treatment. However, a considerable upregulation was still notable for all measured genes (*Nos2*, *Il6*, *Il1b*, *Il12b*, and *Mmp12*) and almost all proteins (IL-1α, IL-6, IL-23, and CCL2) in *Scnn1b*-Tg AMs compared to WT AMs (Fig. 4c, d). Only the chemokine CXCL1 was significantly upregulated in WT AMs at 12 h. At 24 h of LPS treatment, some genes (*Il6, Nos2, Il12b, Mmp12*) and proteins (IL-1α, IL-6, and CCL2) were still significantly upregulated in *Scnn1b*-Tg AMs.

Macrophages obtained from the peritoneum of *Scnn1b*-Tg and WT mice displayed no differences in gene expression after LPS treatment, highlighting a lung-specific macrophage phenotype (Supplementary Fig. 5b). To further test the role of the airway microenvironment on AM responses, we utilized mice with deficiency in cystic fibrosis transmembrane conductance regulator (*Cftr*^−/−^) that causes CF in humans but fails to produce muco-obstructive lung disease in mice^54^. Similar to peritoneal AMs, no differences in LPS responses were detected in *Cftr*^−/−^ AMs compared to WT AMs (Supplementary Fig. 5c). Collectively, these data support an important role of the local microenvironment in proinflammatory responses of macrophages in response to the LPS challenge.

These results suggest a dysregulated and prolonged hyperinflammatory response of AMs from mice with muco-obstructive lung disease. For in-depth analysis of transcriptional and epigenetic changes, we performed paired RNAseq and ATACseq on LPS, and medium treated AMs using the 12 h timepoint to capture the most pronounced alterations in inflammatory responses. Principal component (PC) analysis of the whole transcriptome, as well as chromatin accessibility, separated LPS from medium treated cells in PC1 (explaining ~73% and ~71% of variability, respectively) and the mouse genotype in PC2 (explaining ~7% of the variability in both assays), with one outlier in the RNAseq of WT AMs, treated with LPS (Fig. 4e, f). Functional enrichment analysis of the top 100 genes explaining PC1 of the whole transcriptome included pathways for immune responses, immune activation, and host defense (Fig. 4g). PC2 showed enrichment for interferon-inducible and anti-viral response genes (Fig. 4g), indicating enhanced interferon signaling in *Scnn1b*-Tg AMs compared to WT AMs. Inflammatory regulators, such as LPS, NFKB, IFN-γ, and STAT6, were predicted activators of DEGs in *Scnn1b*-Tg AMs vs. WT AMs after LPS treatment (Fig. S5d). In total, 246 genes were differentially expressed (Supplementary Fig. 5e and Supplementary Data 4). Comparison of ATACseq peaks showed 897 differentially accessible regions in *Scnn1b*-Tg AMs compared to WT AMs (Supplementary Fig. 5f and Supplementary Data 5). Significant motif enrichment in DARs with increased accessibility in *Scnn1b*-Tg AMs was strongly associated with TFs regulating inflammatory responses (e.g., IRF1, IRF2, IRF3, IRF8, NFY, and NFkB-p65, Supplementary Data 6).

Together, these data indicate an immense rearrangement of chromatin accessibility in AMs from the different genotypes upon LPS challenge with consequences on their gene expression. The enhanced transcriptional activation in *Scnn1b*-Tg AMs could relate to differences in chromatin accessibility of regions responsive to LPS. Therefore, we defined LPS-responsive DARs (Fig. 4h and Supplementary Data 7) and compared the accessibility of these regions in *Scnn1b*-Tg AMs and WT AMs upon LPS stimulation (Fig. 4i). This approach showed a significant increase in DAR-accessibility in LPS treated *Scnn1b*-Tg AMs compared to WT AMs (Fig. 4i, upper panel). The identified LPS-responsive DARs exhibited increased accessibility already at baseline level in primary uncultured *Scnn1b*-Tg AMs (Fig. 4i, lower panel), supporting proinflammatory epigenetic priming of AMs in muco-obstructive lung disease.

### Differential TF activity orchestrates LPS responses in *Scnn1b-*Tg AMs

**Fig. 5:**
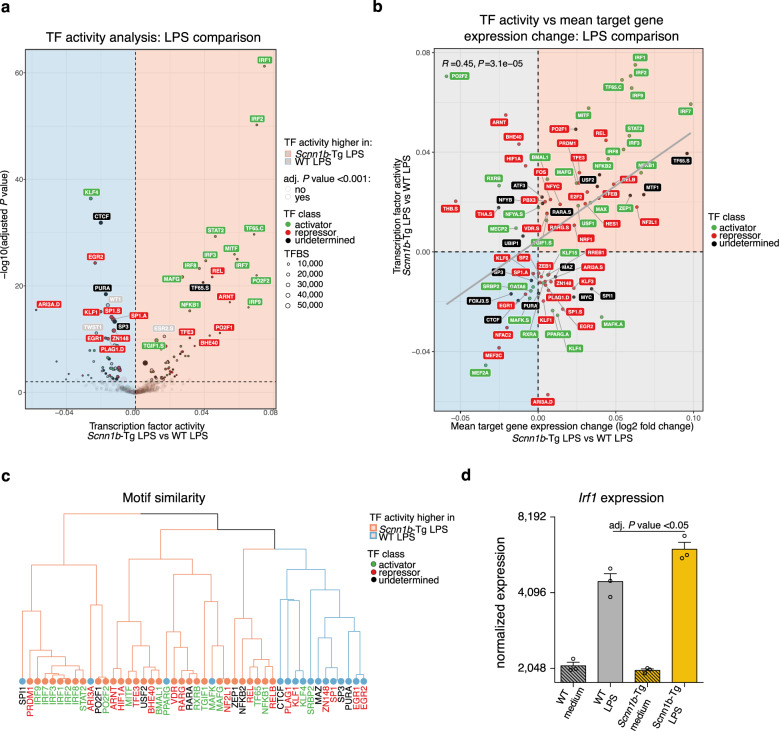
Differential transcription factor activity orchestrates LPS responses in *Scnn1b*-Tg AMs. **a** Differential transcription factor (TF) activity analysis of lipopolysaccharide (LPS) treated *Scnn1b*-transgenic (Tg) vs. wild-type (WT) airway macrophages (AM). A positive weighted mean difference indicates increased TF activity in *Scnn1b*-Tg AMs. Size indicates the number of TF binding sites (TFBS). Green labeled TFs are predicted activators, red labeled TFs are predicted repressors, and black labeled TFs have no direction assigned. **b** Pearson correlation of TF activity of significantly enriched TFs (adjusted (adj.) *P* value < 0.001) in *Scnn1b*-Tg vs. WT AM treated with LPS on the *y*-axis, with mean target gene expression change on the *x*-axis. The gray diagonal represents the linear regression. **c** Clustering of differentially activated TFs (adj. *P* value <10e−6) based on the similarity of their position weight matrixes. Coloring of tree leaves based on TF activity. Coloring of labels defined by TF class. **d** Normalized *Irf1* gene counts obtained by RNA sequencing (RNAseq) (*Scnn1b-*Tg LPS vs. WT LPS) in treated and untreated *Scnn1b*-Tg and WT AMs. Adj. *P* values were determined by DESeq2. The data show mean ± SEM of *n* = 3. Source data are provided as a Source Data file.

The initiation of gene expression is essentially determined by distinct TFs that can bind to their respective motif at accessible chromatin within gene regulatory regions. To evaluate the involvement of specific TFs on hyperinflammatory responses in *Scnn1b*-Tg AMs, we incorporated the RNAseq and ATACseq data from LPS-treated AMs for the prediction of TF activity. Using diffTF^55^, we identified 80 TFs differentially active in LPS treated *Scnn1b*-Tg AMs (Fig. 5a). Of those, 60% showed increased activity in *Scnn1b*-Tg AMs, with 38% and 45% predicted as activators or repressors, respectively. For 17% of the significantly enriched TFs, no clear status was assigned (undetermined). The projected TF activity strongly correlated (Pearson correlation: *R* = 0.45, *P* value = 3.1 × 10^−5^) with the change in mean target gene expression, defined by the presence of a TF motif within the promoter region (Fig. 5b). Same or similar TF motifs are bound by TFs of the same family. Grouping those members of one TF family, using the position weight matrix clustering tool Rsat^56^, resulted in consistent overall activity changes within the same family of TFs (Fig. 5c). The most active TF cluster identified in *Scnn1b*-Tg AMs belonged to the IRF family, including IRF1, IRF2, IRF3, IRF7, IRF8, and IRF9. *Irf1* was also significantly upregulated on the transcriptional level in LPS treated *Scnn1b*-Tg AMs (Fig. 5d). In line with the increased target gene expression of genes carrying an IRF motif in their promoter region. Pearson correlation of the TF expression and the chromatin accessibility of their target regions predicted these TFs as activators compared to regions consisting of other motifs (Supplementary Fig. 6a, upper panel). As further validation, the average chromatin accessibility at putative IRF binding regions was visualized and confirmed increased accessibility for all IRF motif regions in *Scnn1b*-Tg AMs upon LPS treatment (Supplementary Fig. 6a, lower panel).

Similar results were obtained comparing *Scnn1b*-Tg and WT AMs cultured in medium for 12 h, further supporting the hypothesis that AMs from mucus-obstructive airways are primed for enhanced inflammatory response (Supplementary Fig. 6b, c). Although much is known about the activation of the IRF TF family by LPS-mediated TLR4 signaling^57^, little has been reported about its role in macrophages in the muco-obstructive airway microenvironment. Here, we show that the activity of the IRF TF family is enhanced in *Scnn1b*-Tg AMs and associated with an increased expression of hyperinflammatory genes under inflammatory conditions.

### Mucus stimulates immune responses in AMs

**Fig. 6:**
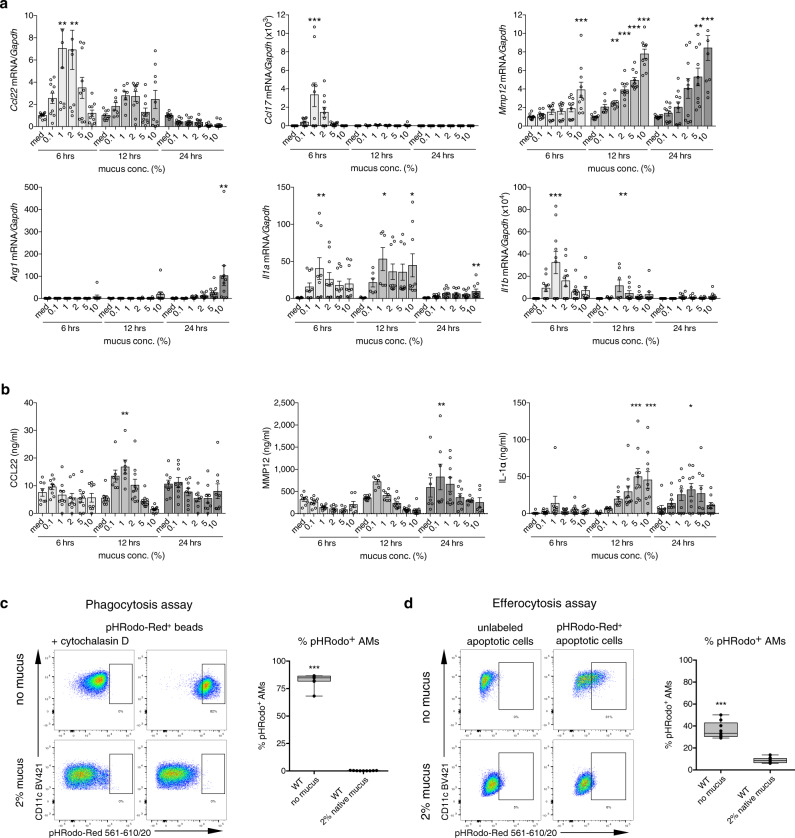
Mucus triggers AM immune responses and functions. **a** Gene expression levels of *Ccl22*, *Cccl17*, *Arg1*, *Mmp12*, *Il1a*, and *Il1b*, and **b** protein expression levels of CCL2, MMP12, and IL-1α in the supernatant of wild-type (WT) airway macrophages (AM) treated with increasing mucus concentrations or medium (med) for 6, 12, and 24 h (hrs). Representative flow cytometry plots and percentage of pHRodo^+^ AMs, depicting **c** phagocytosis and **d** efferocytosis capacities of AMs treated with 2% mucus or medium. Bar plots show mean ± SEM of **a** 6, 24 h: *n* = 10 per group. 12 h: *n* = 9 (med), *n* = 7 (0.1, 1%), *n* = 10 *Ccl22, Ccl17, Mmp12, Arg1, Il1b* (2, 5, 10%), *n* = 9 *Il1a* (2, 5, 10%), and **b** 6 h: *n* = 8 (CCL22, MMP12, med), *n* = 7 (IL-1α, med), *n* = 9 (0.1, 1%), *n* = 9 (CCL22, IL-1α, 2%), *n* = 8 (MMP12, 2%), *n* = 9 (CCL22, IL-1α, 5%), *n* = 6 (MMP12, 5%), *n* = 9 (CCL22, IL-1α, 10%), *n* = 7 (MMP12, 10%). 12 h: *n* = 9 (med), *n* = 7 (CCL22, IL-1α, 0.1%), *n* = 6 (MMP12, 0.1%), *n* = 7 (1%), *n* = 10 (2, 5, 10%). 24 h: *n* = 9 (CCL22, IL-1α, med), *n* = 7 (MMP12, med), *n* = 9 (CCL22, IL-1α, 0.1%), *n* = 7 (MMP12, 0.1%), *n* = 9 (1%), *n* = 8 (CCL2, MMP12, 2%), *n* = 9 (IL-1α, 2%), *n* = 8 (CCL2, IL-1α, 5%), *n* = 6 (MMP12, 5%), *n* = 9 (CCL22, IL-1α, 10%), *n* = 6 (MMP12, 10%). *P value < 0.05; **P value < 0.01; ***P value < 0.001 by one-way ANOVA following Bonferroni´s post hoc test. Box plots indicate the largest value within the 1.5× interquartile range above 75th percentile, 75th percentile, median, 25th percentile, and smallest value within the 1.5× interquartile range below 25th percentile of **c**, **d** *n* = 9 per group. *P value < 0.05; **P value < 0.01; ***P value < 0.001 by Mann–Whitney *U*-test. Source data are provided as a Source Data file.

Various stimuli in the airways can potentially alter the phenotype and functions of macrophages and lead to epigenetic and transcriptional changes. To test whether mucus can activate AMs, we stimulated primary AMs from WT mice with different concentrations and durations of native mucus. We analyzed the expression of selected genes associated with AM plasticity (*Ccl22*, *Ccl17*, *Mmp12*, and *Arg1*) and inflammation in chronic lung diseases (*Il1a* and *Il1b*). All tested genes showed upregulation after mucus treatment (Fig. 6a), similar to changes observed in primary *Scnn1b*-Tg AMs. Upregulation of gene expression for *Ccl17*, *Ccl22*, and *Il1b* was transient with a peak of expression at 6 h. For *Il1a* and *Mmp12*, we observed a sustained upregulation of gene expression at most time points tested and a further concentration-dependent induction for *Mmp12*. Only *Arg1* displayed a delayed induction, with detectable transcripts at 24 h of post-treatment. We observed induction of CCL22 and MMP12 at a concentration of 0.1–10% of native mucus on protein expression level, with CCL22 showing a significant upregulation at 12 h and MMP12 at 24 h of post-treatment (Fig. 6b). IL-1α was significantly upregulated 12 h of post-treatment and showed a concentration-dependent upregulation (Fig. 6b). IL-1β protein was not detectable.

Furthermore, macrophage functions in bacterial and apoptotic cell clearance were assessed post mucus stimulation. For these studies, the corresponding assays used for native AMs (Fig. 6c, d) were adapted for in vitro analysis, and WT AMs were treated with 2% mucus for 12 h. Flow-cytometry analysis revealed a complete impairment of phagocytosis and efferocytosis capacities of AMs (Fig. 6c, d and Supplementary Fig. 7b, d). Collectively, these results show that mucus can directly activate AMs and impairs their key functionalities, an observation with possible implications for disease pathogenesis and treatment.

## Discussion

In this study, we demonstrate that the altered airway microenvironment in muco-obstructive lung disease led to proinflammatory changes in the epigenetic landscape of AMs. We found that *Scnn1b*-Tg AMs are altered in the DNA methylation and chromatin accessibility levels, with changes enriched in promoter and enhancer regions, favoring the binding of TFs involved in macrophage inflammation and polarization. Those changes correlated with the induction of a transcriptional program in AMs from muco-obstructive lungs. The epigenetic modifications in *Scnn1b-*Tg AMs had functional consequences leading to impaired efferocytosis and phagocytosis as well as prolonged hyperinflammatory responses upon LPS challenge. The impaired LPS response of *Scnn1b*-Tg AMs was linked to increased transcription factor activity of the IRF family that controls macrophage polarization, inflammation, and remodeling^36^. Furthermore, we demonstrate that stimulation of WT AMs with mucus induced a transcriptional program reminiscent of the one observed in primary *Scnn1b*-Tg AMs and inhibited their efferocytosis and phagocytosis capacities.

In muco-obstructive lung diseases, such as CF and COPD, AMs reside in different airway niches with specific local environmental signals. Those niches include regions of excess mucus and mucus plugging, which in turn trigger local hypoxia, increased cellular necrosis, apoptosis, infection, and inflammation. Our data show that the sustained exposure to signals provided by this microenvironment of mucus-obstructed airways leads to epigenetic reprogramming of AMs. This manifested in reduced DNA methylation and increased chromatin accessibility of gene regulatory regions and TF-binding motifs with a major role in regulating macrophage activation, plasticity, and inflammation (e.g., NFKB, AP-1, IRF2/3, CEBP, STAT6, ATF3) (Fig. 1). The identified transcription factors possess different transcriptional control of macrophages. NFKB and AP-1 promote inflammatory responses, whereas IRF2/3 and ATF3 are suppressors of macrophage inflammation^36,38,58^. NFKB and AP-1 are also known to promote classical activation of macrophages, while CEBP, STAT6, and ATF3 promote alternative activation^59^. Some of the identified TFs are associated with chronic lung diseases. NFKB showed increased nuclear translocation in AMs from CF patients and is highly expressed in the airways of COPD and asthma patients^60,61^. STAT6 plays a major role in type 2 inflammation, linked to IL-33 induced IL-13 release in the airways^62–64^. Epigenetic modification of macrophages was also shown in AMs from COPD patients, with increased baseline expression of inflammatory markers that persist even after prolonged ex vivo culture^65^. This could be linked to increased acetylation of histones in promoter regions of inflammatory genes^66^. Similarly, AMs from asthmatic patients show increased enzymatic activity of histone acetylases augmented by repressed histone deacetylase activity, eventually leading to increased transcription of inflammatory genes. The enzymatic activity was unchanged in peripheral blood mononuclear cells of asthmatic patients, supporting the role of the local lung environment in AMs function^67^. In line with the epigenetic aberrations in our model, the transcriptional profile of *Scnn1b*-Tg AMs showed coinciding alterations, with significant upregulation of classical (e.g., *Ccl2, Ptgir, Ccl9, Il1a, Marcks, H2-Aa, H2-Eb1*) as well as alternative markers (e.g., *Mmp12, Arg1, Trem2, Ccl22, Ccl17, Ptgs1*) of macrophage activation (Fig. 2). The respective stimuli for classical (IFN-γ and LPS) and alternative (IL-4) macrophage activation were predicted as upstream regulators of the transcriptome and chromatin accessibility changes. The lung microenvironment in *Scnn1b*-Tg mice can support AMs lineage determination in both directions: through increased baseline expression of IL-4, IL-13, and TNF, and a higher bacterial burden^5,64,68^. The presence of AM subtypes in the lungs was substantiated by specific protein expression using high-dimensional flow cytometry, with an increase of marker expression associated with AM activation in *Scnn1b*-Tg mice (Fig. 3). The coexistence of different AM clusters, co-expressing classical and alternative macrophage activation markers, supports a concept in which macrophage phenotypes are a continuum, reflecting cellular adaptation to environmental perturbations. This is in line with studies describing the existence of different macrophage phenotypes in COPD and asthma^69,70^. In a previous report, single-cell transcriptome analysis of COPD patients confirmed the transcriptional plasticity of AMs^71^. This study also depicted the difficulty of interpatient heterogeneity in COPD, emphasizing the importance of mouse models for a basic molecular understanding of muco-obstructive lung diseases.

The disease-associated epigenetic dysregulation had marked functional consequences for AMs. Not only efferocytosis and phagocytosis were reduced, but the treatment of *Scnn1b*-Tg AMs with LPS led to significantly increased cytokine production (Fig. 4). Similarly, a previous study showed increased release of inflammatory mediators of AMs from CF patients treated with LPS^72^. We were able to link the prolonged and hyperinflammatory response with the mucus-obstructed microenvironment rather than mutations in *Cftr*, as *Scnn1b-*Tg macrophages from the peritoneum or AMs from *Cftr*^−/−^ mice responded similarly to WT controls. Airway infections with gram-negative bacteria such as *P. aeruginosa* are frequently found in CF and other muco-obstructive lung diseases, and excessive inflammatory responses are deleterious for host tissue integrity and homeostasis^73,74^. Our integrated analysis of ATACseq and RNAseq of LPS treated AMs revealed the IRF transcription factor family and especially IRF1 as a potential mediator of this process (Fig. 5). Previous studies reported the essential role of IRF1 in LPS-induced acute lung injury^75^, and enhanced IRF1 signaling in AMs from muco-obstructive lungs may contribute to the high morbidity and mortality of patients with CF and COPD with acute pulmonary exacerbations^61,73,74,76^. Recent developments of epigenetic inhibitors targeting the bromodomains and extra terminal domain (BET) family of proteins could modulate the immunoinflammatory response of macrophages in chronic lung diseases^77–79^ and refine therapeutic strategies of muco-obstructive lung diseases.

There are many putative immunomodulatory factors in muco-obstructive lung diseases that may interact with AMs and drive epigenetic reprogramming. One of them is mucus that can contribute to lung pathology and interact through mucin glycans with leukocyte receptors^7,9,80^. Mucus concentrations in the lungs and sputum of muco-obstructive patients are raised, with CF secretions from young adults being approximately three times higher than normal controls^2,81^. Upon stimulation of WT AMs with native mucus, genes reminiscent of the *Scnn1b*-Tg AM phenotype were upregulated in WT AMs (Fig. 6). These findings are consistent with the notion that interactions of mucin glycoproteins with AM surface receptors may be implicated in AM activation in muco-obstructive lung disease. These interactions may regulate AM inflammatory responses, resolution, and homeostasis. In mice, a previous study showed that MUC2 expressed in the gut represses inflammation and induces tolerance in DCs^80^. In line with this, MUC5B interaction with Siglec-F on lung eosinophils induced their apoptosis in allergic inflammation, although an effect on Siglec-F expressing AMs was not observed^7^.

In addition to the immunomodulatory effect, which should be explored in-depth via genome-wide sequencing methods in future studies, an apparent impairment of essential AM functions was observed upon stimulation of WT AMs with mucus. Similar to the comparison of *Scnn1b-*Tg and WT AMs, reductions of both phagocytosis and efferocytosis capacities were observed. Potential discrepancies between the phagocytic activities of primary *Scnn1b-*Tg AMs and mucus-treated WT AMs could be explained by the number of mucus-exposed AMs in the in vivo setting. An inhibitory effect was also reported for the endogenous glycocalyx of cancer cells^82^. Imbert et al. showed that the glycocalyx of macrophages and their targets could constitute a physiological barrier, hindering efferocytosis and phagocytosis. In human lung disease, the efferocytosis and phagocytosis capacities of lung macrophages isolated from patients with COPD and asthma were also shown to be reduced^83–90^. Similarly, a pediatric study involving children with CF demonstrated an impairment of phagocytosis in lung macrophages^14,91^. Reduction in AM functions that are pivotal in maintaining lung homeostasis could contribute to lung pathology and disease progression. In particular, mucus as a critical component of the muco-obstructive microenvironment has the potential to modulate AM phenotype and functions. Therefore, understanding the potential interactions between mucus/mucins and macrophages in health and AM dysfunction in muco-obstructive lung diseases has the potential to guide the development of new therapeutic approaches that may be beneficial for patients with a spectrum of muco-obstructive lung diseases.

In summary, we present a comprehensive study of epigenetic reprogramming of AMs in the in vivo pathogenesis of muco-obstructive lung disease. Our data showed changes associated with a diverse AM phenotype and pathophysiologically relevant changes in their immune responses. Identifying and understanding the mechanisms of AM interactions with the airway microenvironment and its contribution to the epigenetic reprogramming of AMs will guide the development and improvement of immunomodulatory therapies.

## Methods

### Mice

All animal studies were approved by the animal welfare authorities responsible for the University of Heidelberg (Regierungspräsidium Karlsruhe, Karlsruhe, Germany). *Scnn1b*-Tg mice on a C57BL/6 background and gut-corrected *Cftr*^*−/−*^ mice (Cftrtm1Unc Tg(FABPCFTR)) on C57BL/6 background^92^ were bred in-house under specific pathogen-free conditions and genotyped as previously described^93^. For experiments involving FACS followed by downstream sequencing, 6-week-old female mice were used. For all other experiments, gender-matched 6-week-old mice were used. WT littermates were used as control animals. Mice were housed at room temperatures of 22 ± 2 °C with 50–60% humidity and kept on a 12/12 h light/dark cycle with continuous access to food and water.

### Isolation of AMs by lavage

Mice were anesthetized by intraperitoneal injection of 120 mg/kg Ketamine and 16 mg/kg Xylazine (Sigma-Aldrich, Darmstadt, Germany) and exsanguinated. Primary AMs were collected by flushing the lungs three times with 800 μl of PBS supplemented with 5 mM EDTA (Sigma-Aldrich). The lavage was repeated twice. For ex vivo treatment studies, 3–4 lavages were pooled to maximize the obtained cell numbers.

### Isolation of peritoneal macrophages by lavage

Mice were euthanized by cervical dislocation. Primary peritoneal macrophages were collected by carefully injecting 10 ml of PBS supplemented with 5 mM EDTA (Sigma-Aldrich) into the peritoneum and aspiration of the fluid.

### Culture and treatment of primary macrophages

Collected cells were seeded into a flat-bottom 96-well plate in complete DMEM (1 g/l d-Glucose, l-Glutamine, Pyruvate; Gibco, Dreieich, Germany) containing 10% heat-inactivated FCS (Gibco), 1× penicillin-streptomycin (Gibco) 2 mM l-glutamine (Gibco) to a final count of 1 × 10^5^ AMs/well, for LPS treatment, according to cell composition determined by cytospin. For baseline gene expression, all cells were seeded. After 1 h incubation at 37 °C, 5% CO_2_, the non-adherent cells were gently washed from the plate with prewarmed PBS. For baseline gene expression analysis, adherent cells were resuspended in 200 μl Trizol (Thermo Fisher Scientific GmbH, Dreieich, Germany). For LPS treatment, adherent cells were exposed to 100 ng/ml LPS from *P. aeruginosa* (Sigma-Aldrich) for 6, 12, and 24 h or complete DMEM as control. For mucus treatment, AMs were exposed to different concentrations of bovine submaxillary gland mucus (Merck Millipore, Darmstadt, Germany). Supernatants were collected, and the cells were resuspended in 200 μl Trizol for RNA extraction.

### Primary mouse tracheal epithelial culture (mTEC)

Mice were anesthetized by intraperitoneal injection of 120 mg/kg Ketamine and 16 mg/kg Xylazin (Sigma-Aldrich), exsanguinated, and the trachea was removed. Excised tracheas were cut open, washed in DMEM/F-12 (Gibco) containing 1% penicillin-streptomycin (Gibco), and transferred to 20 ml dissociation medium containing 1.4 mg/ml protease E (Sigma-Aldrich) and 0.1 mg/ml DNase I (Roche Diagnostics GmbH, Mannheim, Germany) and incubated overnight at 37 °C. The enzymatic reaction was stopped with heat-inactivated FCS (Gibco), cell-suspension was filtered through a 100 μm cell strainer (BD Biosciences, Heidelberg, Germany), resuspended in DMEM/F-12 supplemented with heat-inactivated FCS (Gibco), 4.4 μg/ml insulin (human recombinant zinc; Gibco), and 0.1 mg/ml primocin (InvivoGen, Toulouse, France), and seeded into bacterial culture dishes. After 2 h incubation at 37 °C non-adherent epithelial cells were seeded on transwell filters (Costar, Sigma-Aldrich) coated with collagen from the human placenta (Sigma-Aldrich). Medium for submerged culturing (mTEC/Plus) and culturing in an air-liquid interface (ALI, mTEC/SF) was adapted by Horani et al.^94^. The basic medium for mTEC/Plus and mTEC/SF medium consists of DMEM/F-12 (Gibco) supplemented with 15 mM HEPES (Carl Roth GmbH & Co. KG, Karlsruhe, Germany), 3.6 mM NaHCO3 (ApplieChem GmbH, Darmstadt, Germany), 4 mM L-glutamine (Gibco) and 1x penicillin-streptomycin (Gibco). Medium for submerged culture was prepared as followed; basic medium was supplemented with 10 μg/ml insulin (human recombinant zinc, Gibco), 5 μg/ml transferrin (Sigma-Aldrich), 25 ng/ml recombinant human epidermal growth factor (EGF, Gibco), 30 μg/ml bovine pituitary gland extract (BPE, Sigma-Aldrich), 5% heat-inactivated FCS (Gibco). Medium for ALI culturing was prepared as followed; basic medium was supplemented with 5 μg/ml insulin (human recombinant zinc, Gibco), 5 μg/ml transferrin (Sigma-Aldrich), 5 ng/ml EGF (Gibco), 30 μg/ml BPE (Sigma-Aldrich) and 1 mg/ml BSA (SERVA Electrophoresis GmbH, Heidelberg, Germany) reconstituted in HBSS (Gibco). Retinoic acid (RA, Sigma-Aldrich) must be added freshly to mTEC/Plus and mTEC/SF medium prior to use (final conc. 5 × 10^−8^ M). After three days of submerged culture, the medium was changed, and on day five, transepithelial resistance was measured. When a value of 1000 Ω/cm^2^ was reached, cells were transferred to ALI culture conditions using mTEC/SF medium plus RA. Medium change and surface wash with PBS every other day.

### Immunocytochemistry

The cells were fixed with 4% paraformaldehyde (Otto Fischar GmbH & Co. KG, Saarbrucken, Germany) for 10 min, permeabilized with 0.2% Triton X-100 (ApplieChem GmbH) for 8 min and blocked with 1% BSA (SERVA Electrophoresis GmbH) for 15 min. Staining was performed with rabbit anti-mouse-SCNN1B (1:200, kindly provided by Prof. Dr. C. Korbmacher, University Erlangen-Nuremberg), rat anti-mouse-MerTK (1:20; R&D Systems Inc., Wiesbaden, Germany), and mouse anti-mouse-acetylated-α-tubulin (1:200; Life Technologies, Dreieich, Germany) for 60 min at 37 °C. Followed by staining with the respective secondary F(ab’)_2_ fragment goat anti-rabbit IgG (AF647, 1:200, Life Technologies), F(ab’)_2_ fragment goat anti-rat IgG (AF488, 1:300, Life Technologies) and F(ab’)_2_ fragment goat anti-mouse IgG (AF488, 1:200, Life Technologies) and Hoechst 33258 (1:20,000, Life Technologies), for nuclei staining, for 2 h at RT. Pictures were acquired with a Leica TCS SP8 (Leica Microsystems, Wetzlar Germany) and analyzed using the LASX v3.5.19976 and FIJI v2.0.0.

### FACS sorting of AMs for sequencing

One ml dispase (BD Biosciences) was intratracheally instilled into the lungs and plugged inside by instillation of 300 μl low melting agarose (1%). When agarose is solid, the whole lung was extracted, including the tracheobronchial tree, put into 2 ml dispase, and left for 35 min at RT. The reaction was stopped with complete DMEM, lungs were mashed with a plunger, the tracheobronchial tree was removed, and the cell suspension was put through a 100 μm cell strainer (BD Biosciences). After red blood cell lysis (RBC lysis buffer, eBiosciences, Dreieich, Germany), the cell suspension was enriched by magnetic bead separation using CD45^+^ magnetic beads, according to the manufacturer’s instructions (Miltenyi Biotech, Bergisch Gladbach, Germany). After positive selection, cells were counted, and antibody-concentration was used according to obtained cell numbers, as prior determined by titration (see Supplementary Table 1). Cells were incubated for 5 min with purified rat IgG2b anti-mouse CD16/CD32 receptor antibody (BD Biosciences, Heidelberg, Germany) in FACS buffer containing 1% BSA (SERVA Electrophoresis GmbH, Heidelberg, Germany) and 5 mM EDTA (Sigma-Aldrich), following staining with fluorochrome-conjugated antibodies against mouse CD45.2, Siglec-F, and CD11c. Samples were stained with 7-AAD (0.25 μg/ml per 1 × 10^6^ cells; Biolegend, London, UK) 5 min prior to sort, for dead cell exclusion. Sorting was performed at the EMBL Flow Core Facility, Heidelberg, Germany. AMs were sorted using a standard BD Fusion equipped with 100 mW 405 nm, 100 mW 488 nm, 80 mW 561 nm, 80 mW 640 nm lasers and an ND2.0 filter in front of the FSC photodiode, a nozzle size of 100 μm, and corresponding BD FACSFlow sheath pressure of 20 psi, matched with a transducer frequency of 32 kHz. Input pressure was adjusted to ensure that an event populated every fifth to the sixth drop. A purity check of sorted cells was performed on selected samples from each run, confirming purities ranging from 95 to 99%. Sorted AMs were either immediately processed or treated with 100 ng LPS for 12 h for nucleic acid isolation and sequencing. FMO controls were used as gating controls when low spectral overlap did not skew the populations. This was the case for all chosen colors, and antibody matches in our experimental setup.

### Flow cytometry staining for surface marker expression

After lung perfusion with PBS, the whole lung was digested with 1 mg/ml Collagenase D (Roche Diagnostics GmbH, Mannheim, Germany) and 30 μg/ml DNAse I (Roche Diagnostics GmbH) to obtain a single-cell suspension. After 1 h incubation at 37 °C on a shaker, digested lungs were mechanically put through a 100 μm cell strainer (BD Biosciences, Heidelberg, Germany), red blood cells were lysed (RBC lysis buffer, eBiosciences), and two million cells were used for staining. Cells were incubated for 5 min with purified rat IgG2b anti-mouse CD16/CD32 receptor antibody (BD Biosciences) in FACS buffer containing 1% BSA (SERVA Electrophoresis GmbH), 5 mM EDTA (Sigma-Aldrich), and 0.05% sodium azide (Sigma-Aldrich), following staining with fluorochrome-conjugated antibodies against mouse CD45.2, Siglec-F, CD11b, CD11c, CD64, MerTK, CD200R, CD206, CD163, CD38, CD86, CD68, MHCII, MGL2, CLEC7A, and CD209A (see Supplementary Table 1) in brilliant stain buffer (BD Biosciences) for 25 min at 4 °C. This was followed by 30 min staining at 4 °C with live/dead fixable dye APC-eFlour780 (1:1000; eBiosciences). Samples were acquired on a standard 405/488/561/640 nm laser engine CYTEK Aurora (Cytek biosciences, Freemont CA, USA). Data were analyzed using FACSDiva v8.0 and FlowJo v10.7 (BD Biosciences). FMO controls were used as gating controls when low spectral overlap did not skew the populations. This was the case for all chosen colors and antibody matches in our experimental setup.

### Analysis of surface marker expression

Flow cytometry data were preprocessed using FACSDiva software and FlowJo 10.4 (BD Biosciences). Frequency of AMs (CD45.2^+^ CD64^+^ MerTK^+^ CD11c^+^ SiglecF^+^) positive for macrophage activation markers was assessed with FlowJo 10.4. Next, leukocytes were defined based on the expression of CD45.2^+^. Fluorescence intensities were exported for further analysis according to the workflow described by Nowicka et al.^95^. In short, fluorescence intensities were transformed with the arcsine-square-root transformation, and cell clustering was performed with the R packages FlowSOM^96^ v1.18.0 and ConsensusClusterPlus^97^ v1.50.0. 20 meta clusters were defined that were assigned to cell types based on the expression of specific surface markers: Airway macrophages: CD45.2^+^ CD64^+^ MerTK^+^ CD11c^+^ SiglecF^+^; Interstitial macrophages: CD45.2^+^ CD64^+^ MerTK^+^ MHCII^+^ SiglecF^−^; Dendritic cells: CD45.2^+^ CD64^−^ MerTK^−^ SiglecF^−^ MHCII^+^ CD11c^+^; Neutrophils: CD45.2^+^ CD64^−^ MerTK^−^ CD11b^+^ SiglecF^−^ MHCII^−^; Eosinophils: CD45.2^+^ CD64^−^ MerTK^−^ CD11b^+^ SiglecF^+^; B-cells: CD45.2^+^ CD64^−^ MerTK^−^ SiglecF^−^ MHCII^+^ CD38^+^. Visual representation with UMAP was performed by subsampling 5000 cells per sample and the R package umap^98^ v0.2.4.1. To perform differential surface marker expression, stratified by cell types, a linear mixed model was applied, including the flow cytometry experiments’ date as a covariate. Differentially expressed surface markers were defined by an adjusted *P* value < 0.05. Similarly, differential cluster abundancy was performed on the frequency of AM sub-clusters by applying a linear mixed model including the flow cytometry experiments’ date as a covariate. The differential abundance of AM sub-clusters was defined by an adjusted *P* value < 0.05.

### Flow cytometry staining for efferocytosis and phagocytosis assay

Mouse LA4 ATCC CCL-196 cells (5 × 10^5^ cells/ml; ATCC, Wesel, Germany) were incubated for 3 h with 1 μM staurosporine (Enzo Life Sciences (ELS) AG, Lausen, Switzerland) to induce apoptosis. Apoptotic cells were labeled with an equimolar solution of Annexin V-Biotin (Biolegend) and pHRodo Red Avidin (Thermo Fisher Scientific GmbH, P35362) in Annexin V staining buffer (Biolegend), according to manufacturer’s instructions, and left for 15 min at RT. A total of 1 × 10^6^ apoptotic cells resuspended in 50 μl PBS, were intratracheally administered into mice anesthetized with isoflurane. After 2–3 h, mice were sacrificed, and airway macrophages were isolated by lavage of the lungs. For phagocytosis, cells were obtained by lavage and incubated for 1 h with or without the phagocytosis inhibitor Cytochalasin D (10 μM, Sigma-Aldrich) in complete DMEM, followed by 1 h incubation with pHRodo Red *E. coli* Bioparticles (P35361), according to manufacturer´s instructions (Thermo Fisher Scientific GmbH). For flow cytometry, cells were incubated for 5 min with purified rat IgG2b anti-mouse CD16/CD32 receptor antibody (BD Biosciences) in FACS buffer containing 1% BSA (SERVA Electrophoresis GmbH), 5 mM EDTA (Sigma-Aldrich), and 0.05% sodium azide (Sigma-Aldrich), following staining with fluorochrome-conjugated antibodies against mouse Siglec-F, CD45.2 and CD11c (see Supplementary Table 1) for 25 min at 4 °C in the dark. After washing, cells were incubated for 30 min at 4 °C with Live/dead fixable dye APC-eFlour780 (1:1000; eBiosciences). Samples were acquired on a BD LSRFortessa equipped with 20 mW 355 nm, 50 mW 405 nm, 50 mW 488 nm, 50 mW 561 nm, 40 mW 640 nm lasers, and an ND1.0 filter in front of the FSC photodiode. Internalized LA4 cells and *E. coli* Bioparticles in macrophages were tracked with the pHrodo dye. Data were analyzed using FACSDiva v8.0 and FlowJo v10.7 (BD Biosciences).

For phagocytosis and efferocytosis assays involving treatment with bovine submaxillary gland mucus (Merck Millipore), AMs were isolated by lavage, and 100,000 cells were seeded into 96-well or 48-well plates, respectively. Plates were coated with Poly(2-HEMA) (Sigma-Aldrich) prior to the experiments to ease the release of AMs from the plate for flow cytometry staining. AMs were exposed to 2% mucus for 12 h in the incubator.

For efferocytosis, LA4 ATCC CCL-196 cells were treated as described above, and 200,000 apoptotic LA4 cells were added to AMs and incubated for 2 h in the incubator. For phagocytosis, cells were incubated for 1 h with or without the phagocytosis inhibitor Cytochalasin D (10 μM) in complete DMEM, followed by 1 h incubation with pHRodo Red *E. coli* Bioparticles, according to manufacturer´s instructions (Thermo Fisher Scientific GmbH). The staining procedure for flow cytometry was conducted as described above. However, staining was performed using the fluorochrome-conjugated antibodies against mouse Siglec-F and CD11c (see Supplementary Table 1). Prior to acquiring the data, the DNA dye Draq7 (1:50, Biostatus, Shepshed, United Kingdom) was added to the samples for 5 min at RT for life/dead discrimination. Samples were acquired on a BD LSRFortessa equipped with 20 mW 355 nm, 50 mW 405 nm, 50 mW 488 nm, 50 mW 561 nm, 40 mW 640 nm lasers, and an ND1.0 filter in front of the FSC photodiode. Internalized LA4 cells and *E. coli* Bioparticles in macrophages were tracked with the pHrodo dye. Data were analyzed using FACSDiva software and FlowJo 10.4 (BD Biosciences). FMO controls were used as gating controls when low spectral overlap did not skew the populations. This was the case for all chosen colors and antibody matches in our experimental setup.

### RNA isolation and gene expression analysis for qPCR

RNA extraction was performed using Trizol (Thermo Fisher Scientific GmbH) and RNeasy Micro Kit (74004, Qiagen, Düsseldorf, Germany), according to the manufacturer’s instructions. Reverse transcription was used to generate cDNA (Superscript III Reverse Transcriptase, Invitrogen, Dreieich, Germany). To quantify mRNA levels in AMs, real-time quantitative PCR was performed. For mRNA analysis, the following assays, supplied by Thermo Fischer Scientific were used: *Nos2, Il1b, Il12b, Mmp12, Arg1, Ccl22, Ccl17, Cd86, Cxcr1, Trem2, Ptgs1, Ptgir, Anpep, Igf1, Igf2bp3* together with *Gapdh* (primer limited) in duplex runs. For *Il6*, the following primer and probes were used: fwd, 5′-GAGGATACCACTCCCAACAGACC-3′, rev, 5′-AAGTGCATCATCGTTGTTCATACA-3′; probe, 5′-FAM-CAGAATTGCCATTGCACAA-TAMRA-3′. For *Tnf*, the following primer and probes were used: fwd, 5′-CATCTTCTCAAAATTCGAGTGACAa-3′; rev, 5′-TGGGAGTAGACAAGGTACAACCC-3′; probe 5′-FAM-CACGTCGTAGCAAAC-3′ (Eurofins Genomics GmbH, Ebersberg, Germany); (Supplementary table 2). Results were normalized to levels of *Gapdh* as a reference gene, and fold induction was calculated. Analyses were performed using 7500 Real-Time PCR System SDS Software (Applied Biosystems, Dreieich, Germany).

### Chemokine and cytokine detection

Supernatants obtained from medium and LPS treated AMs were analyzed for IL-6 (558301), IL-1α (560157), IL-23 p19/p40 (562575), TNF (558299), CCL2 (558342), CCL3 (558449), and CXCL1 (558340) by cytometric bead array (BD Biosciences) according to manufacturer’s instructions. Samples were measured on a BD FortessaLSR and quantified according to a standard curve using the BD Cytometric Bead Array FCAP Array Software v3 (BD Biosciences). The supernatants obtained from AMs treated with mucus were analyzed with customized Luminex Assay for CCL22, MMP12, IL-1α, and IL-1β (LXSAMSM-04, R&D Systems), and acquired on a Luminex 200 (Thermo Fisher Scientific). Data were analyzed using Bio-Plex Manager 6.2 software.

### Nucleic acid isolation for sequencing

DNA and RNA were isolated using TRIzol reagent (Thermo Fisher Scientific GmbH) following AllPrep DNA/RNA Mini Kit (80204, Qiagen) purification, according to the manufacturer’s instructions. DNAse I (RNase-free DNAse set, Qiagen) digestion was performed for RNA isolation. Proteinase K digestion was performed for RNA and DNA isolation (Puregene Proteinase K, Qiagen). Prior to library preparation, DNA and RNA quality was determined by Agilent Bioanalyzer (Agilent Technologies Germany GmbH & Co. KG, Waldbronn, Germany) and quantified by Qubit fluorometry (Thermo Fisher Scientific GmbH). All RNA samples reached an RNA integrity number (RIN) > 8.5.

### tWGBS library preparation

The generation of tWGBS libraries followed an established protocol^99^ starting with the tagmentation of 20 ng genomic DNA in a 20 µl reaction mix at 55 °C for 8 min. After the addition of 15 µl 5 M guanidinium thiocyanate, the DNA was purified with HighPrep beads (Gaithersburg, MD, USA). Oligonucleotide replacement and gap repair were done in a 20 µl mix at 37 °C for 30 min followed by another bead-mediated purification. Bisulfite treatment was done with the EZ DNA Methylation kit (Zymo, Irvine, CA, USA) using 16 times cycling at 50 °C for 60 min and 95 °C for 15 s. Per sample, four differently barcoded tWGBS libraries were generated under real-time conditions to monitor amplification using a program of initial melting at 95 °C for 3 min followed by cycling with 95 °C for 20 s, 62 °C for 15 s, and 72 °C for 40 s. After bead-mediated purification, libraries were quantified and pooled in equimolar amounts with a final concentration of 10 nM. Each pool was sequenced at the DKFZ Genomics and Proteomics Core Facility using paired-end, 125 bp, on one lane of a HiSeq2000 v4 sequencer (Illumina, San Diego, CA, USA).

### ATACseq library preparation

ATACseq libraries were prepared according to the Omni-ATAC protocol^100^ with minor modifications. In short, 50,000 viable cells were pelleted and washed in PBS. Subsequently, nuclei were isolated using cold lysis buffer containing 0.01% Digitonin (Sigma-Aldrich), 1% NP40 (Genaxxon Bioscience, Ulm, Germany), and 0.1% Tween-20 (Sigma-Aldrich). Resuspension of the nuclei was done in ATAC washing buffer containing 10 mM Tris-HCl, pH 7.4 (Sigma-Aldrich), 10 mM NaCl (Sigma-Aldrich), and 3 mM MgCl_2_ (Sigma-Aldrich). The tagmentation reaction was performed in 2× transposition buffer containing 20 mM Tris-HCl, pH 7.6, 3 mM MgCl_2_, and 20% Dimethyl Formamide (Sigma-Aldrich) and the addition of 2.5 µl of Tagment DNA Enzyme 1 (Illumina) and rotating the mixture at 1000 rpm for 30 min at 37 °C. The reaction was stopped by the addition of 20 µl of 5 M Guanidinium thiocyanate (Sigma-Aldrich). Transposed chromatin was then purified using 30 µl of AMPure XP beads (Beckman Coulter, Brea, CA, USA) and 110 µl of PEG buffer containing 2.5 M NaCl and 20% PEG 8000 (Sigma-Aldrich). Library amplification was separated in a two-step procedure. First, 25 µl of NEBNext High Fidelity 2x Master Mix (NEB, Ipswich, MA, USA), 0.8 µl of 10 µM Custom Nextera PCR Primer 1, and 0.8 µl of 10 µM Custom Nextera PCR Barcode were added to 25 µl of the transposed DNA, using the following program: 5 min at 72 °C, 30 s at 98 °C, 5 cycles of 10 s at 98 °C, 30 s at 63 °C, and 1 min at 72 °C, and finally, 1 min at 72 °C. Five microliter of the pre-amplified PCR mixture were used to determine how many additional cycles were needed to reach sufficient amplification of each library. For this, Sybr Green (Thermo Fisher Scientific GmbH) was added, and qPCR was performed with a light cycler instrument (Roche Diagnostics GmbH) and the following program: 30 s at 98 °C, 20 cycles of 10 s at 98 °C, 30 s at 63 °C, 1 min at 72 °C, and, finally, 1 min at 72 °C. In a second step, the previously identified PCR cycles were applied to the remaining pre-amplified mixture. The libraries were then purified with a left-sided size selection applying 1.4× of AMPure XP beads (Beckman Coulter) and finally resuspended in 1× elution buffer (Qiagen). Fragment size distribution was checked by Agilent Bioanalyzer (Agilent Technologies Germany GmbH & Co. KG), and concentrations were quantified by Qubit fluorometry (Thermo Fisher Scientific GmbH). Sequencing was performed in multiplexes at the DKFZ Genomics and Proteomics Core Facility using the High Seq 2000 v4 paired-end 125 bp platform (Illumina).

### RNAseq library preparation

For the paired-end baseline as well as LPS- and medium-treated replicates of *Scnn1b*-Tg AMs and WT AMs, sequencing libraries were prepared by the DKFZ Genomics and Proteomics Core Facility from total RNA using the SMART-Seq v4 Ultra Low Input RNA Kit (634891, Takara, Saint-Germain-en-Laye, France) according to the manufacturer’s instructions. For sequencing, samples were sequenced on a High Seq 2000 v4, paired-end, 125 bp platform (Illumina). Single-end baseline replicates were prepared by the EMBL Genomics Core Facility from total RNA using the NEBNext Ultra II Directional RNA Library Prep Kit Illumina (E7760L, New England Biolabs GmbH, Frankfurt am Main, Germany). Sequencing was performed on a Next Seq 500, single-end, 75 bp platform (Illumina).

### tWGBS data processing

Raw reads were processed using Trimmomatic^101^ v0.36 and aligned against the mouse reference genome mm10 using bwa mem^102^ v0.7.8 with default parameters, except for invoking “*-T 0*”. Afterward, alignment duplicates were marked by applying *Picard* MarkDuplicates (http://broadinstitute.github.io/picard) v1.125. Methylation calling was performed with *MethylDackel* (https://github.com/dpryan79/MethylDackel) v0.3.0. According to M-bias plot quality control, the five base pairs at the two ends of the reads were excluded from methylation calling. All samples used for downstream analysis had a bisulfite conversion rate of above 98%, and more than 95% of all reference CpG were covered. In total, each *Scnn1b*-Tg and WT group of replicates had a genome-wide CpG coverage of at least 20×.

### ATACseq data processing

Processing of the ATACseq reads was performed using the ENCODE ATACseq pipeline kundajelab/atac_dnase_pipelines v0.3.0 with default parameters^103^. As a reference, genome mm10 was used. Each baseline replicates achieved a minimum of 50 million non-duplicated, non-mitochondrial reads. The irreproducible discovery rate was less than two for each *Scnn1b*-Tg and WT group of replicates. The fraction of reads in called peaks was above 0.5.

### RNAseq data processing

RNAseq data were processed with the nf-core RNAseq pipeline^104^ v1.2. Default parameters were used unless mentioned otherwise. Sequences were aligned to the mouse reference genome mm10 by applying the software HISAT2, with *-unstranded* option. For single-end data, the *-singleEnd* option was applied. Transcripts were assembled using StringTie and gene code gene annotation release M20^105^. Gene counts were generated with Stringties prepDE.py script (setting: -eb).

### Differential methylation analysis

The bsmooth algorithm of bsseq^106^ v1.20.0 was used to smooth the methylation profiles of all samples with default parameters. DMRs in a pair-wise comparison between *Scnn1b*-Tg and WT AMs were called with DSS^107^ v2.32.0. Regions with at least three CpGs, a minimum length of 50 bp, a minimum delta of 0.1, and a Benjamin–Hochberg corrected *P* value < 0.05 were selected.

### Differential accessibility analysis

Differential accessibility analysis was performed using the R package DiffBind^108^. In short, a common peak set was identified by the presence of a peak in at least two samples. As a method of differential analysis, edgeR was applied^109^. For the LPS treatment experiment, the date of library preparation was included as a blocking factor to adjust for batch effects. To define the LPS treatment effect in Fig. 4h, count values were extracted for every consensus peak, and a multi-factor design including the batch, genotype, treatment, and interaction term (~ batch + genotype + treatment + genotype:treatment) was constructed using DESeq2. The treatment covariate was extracted using the following contrast: c (“treatment”, “medium”, “LPS”). LPS responsive regions with an adjusted *P* value < 0.05 and a log2 fold-change >2 were considered as significantly more accessible in LPS vs. medium treated samples. Annotation of all differentially accessible regions was performed with the R package ChIPseeker v1.22.1^110^ and TxDb.Mmusculus.UCSC.mm10.knownGene^111^.

### Differential gene expression analysis

For the identification of differentially expressed genes, the R package DESeq2 v1.26.0 was used^112^. For group-wise comparison of baseline replicates, the littermate was included in the design formula to adjust for batch effects. For the LPS stimulation experiment, the date of library preparation was included. The adjusted *P* value, as well as log2 fold change required to fulfill statistical significance, is mentioned in the figure legends.

### Deconvolution of RNAseq data

Deconvolution of bulk RNAseq with single-cell references was done with the R library MuSiC^28^ v0.1.1. Processed and annotated RNAseq data from the single-cell atlas of the aging lung^29^ and single-cell atlas of inflammatory airspace macrophages^30^ were kindly provided by the authors. Only macrophage and monocyte clusters were used for the deconvolution with the single-cell atlas of the aging lung. All clusters were used for cell type estimation with the single-cell atlas of inflammatory airspace macrophages.

### Enrichment of gene regulatory regions

The R package LOLA was used for testing overlap and enrichment of DMRs and DARs with multi-cell gene regulatory regions from Ensembl^113,114^. DMRs and DARs with an FDR < 0.05 were stratified in hypomathylated and hypermethylated as well as increased and decreased accessibility in *Scnn1b*-Tg vs. WT AMs, respectively. Enrichment was performed against a random background for DMRs or the common peak set identified with DiffBind for DARs^108^.

### Hierarchical clustering

For hierarchical cluster analysis of DNA methylation, Manhattan distance of all DMRs was calculated, and complete linkage clustering was performed. Similarly, for chromatin accessibility, Euclidean distance of TMM normalized counts of each DAR was used^108,115^. For gene expression, regularized log-transformed gene counts were generated using DESeq2^112^ and applied to batch removal with the limma function *removeBatchEffect*^116^. *z*-Scaled values were used to calculate Euclidean distance that was applied for clustering using Ward’s method.

### Motif analysis of DMRs and DARs

For the analysis of enriched DNA motifs, the command line tool Homer was used^117^. For this, all DMRs and DARs with an FDR < 0.05 were stratified in hypo- and hypermethylated or opened and closed regions, respectively. Homer was used with the option *-size given*.

### Upstream regulator analysis with Ingenuity Pathway Analysis

Upstream regulator analysis with the prediction of activation states was done with QIAGEN’s Ingenuity Pathway Analysis, according to the developer’s manual. The analysis is based on prior knowledge of expected effects between transcriptional regulators and their target genes stored in the Ingenuity Knowledge Base. For upstream regulator analysis of chromatin accessibility, DARs were annotated with the R package ChIPseeker^110^ and TxDb.Mmusculus.UCSC.mm10.knownGene^111^. Log2 fold changes as well as adj. *P* values were used for Ingenuity Pathway Analysis.

### Gene set enrichment analysis

GSEA against custom gene sets was performed with the R package clusterProfiler^118^ v3.12.0. For this, genes were ordered according to their log2 fold change, and the fgsea algorithm was applied. Custom gene sets relevant to lung disease and cellular physiology were obtained from Saini et al.^47^.

### Overrepresentation analysis of pathways and gene ontologies

Enrichment analysis of pathways and gene ontologies was performed using the web tool Metascape^119^. For Supplementary Fig. 2, all differentially expressed genes with an adjusted *P* value < 0.1 were used for enrichment analysis. For Fig. 4g, the variable loadings of PC1 and 2 were extracted and ordered by their absolute value. The top 100 genes of PC1 and 2 were selected and further used for overrepresentation analysis with the web tool Metascape^119^.

### Principal component analysis

PCA was performed with the base R function *prcomp*. For expression data, batch-removed regularized log-transformed values were used. In the case of ATACseq data, log-transformed normalized counts per consensus peaks were generated with the R package DESeq2 and the function *rlog*^112^.

### Profile and locus plots

Profile plots were generated with the R package peakseason (https://github.com/PoisonAlien/peakseason). *P* values were determined with students’ *t*-test after the normal distribution was verified by a Shapiro test. Locus plots were generated using the R package gviz v1.30.3^120^.

### Transcription factor activity analysis

To assess differential transcription factor activity, diffTF v 1.3.3 was applied to the ATACseq and RNAseq data^55^. DiffTF was used in analytical mode with default parameters, comparing *Scnn1b*-Tg and WT AMs treated with LPS or medium, respectively. As a reference, 442 mouse transcription factors with in silico predicted transcription factor binding sites, based on the HOCOMOCO 10 database, were applied^121^. Gene expression data of matching samples were incorporated to identify the transcription factor class (activator, repressor, or undetermined). Mean target gene expression was evaluated for transcription factors with an adj. *P* value < 0.001, by averaging the expression log2 fold change for each gene with the respective transcription factor motif less than 1500 bp away from the transcriptional start site. Annotation of the transcription factor binding sites was performed as described above.

### Clustering of transcription factors based on position weight matrix similarity

To define the similarity of differentially active TF (adjusted *P* value < 0.001) based on their position weight matrix (PWM), the clustering results of RSAT were used^56^.

### Statistical analysis

Statistical analyses were performed using R v3.6^122^ or GraphPad Prism 6 (GraphPad Software Inc., San Diego, USA). Two-group comparisons were performed using unpaired, two-tailed Mann–Whitney *U*-test, and multi-group comparisons were performed using one-way ANOVA with Tukey´s or Bonferroni´s multiple comparison test. *P value < 0.05; **P value < 0.01; ***P value < 0.001; ND, not detectable.

### Reporting summary

Further information on research design is available in the Nature Research Reporting Summary linked to this article.

## Supplementary information

Supplementary Information

Description of Additional Supplementary Files

Supplementary Data 1

Supplementary Data 2

Supplementary Data 3

Supplementary Data 4

Supplementary Data 5

Supplementary Data 6

Supplementary Data 7

Reporting Summary

## Data Availability

The sequencing data have been deposited in the NCBI Gene Expression Omnibus (GEO) under the primary accession number GSE154808. The reference genome was acquired from ENCODE. Gen code gene annotation release M20 was downloaded from Gencode^105^. In silico predicted transcription factor binding sites were acquired from the HOCOMOCO 10 database^121^. To cluster differentially active transcription factors, the clustering results of RSAT were applied^56^. Furthermore, custom gene sets from Saini et al. were used for gene set enrichment analysis^123^. Source data are provided with this paper.

## Source data

Source Data

## Code availability

Data analysis is based on publicly available software listed in the “Methods” section.
