# Supplementary material for: Epigenetic reprogramming of airway macrophages promotes polarization and inflammation in muco-obstructive lung disease
Source: Nat Commun. 2021 Nov 11;12:6520. doi: 10.1038/s41467-021-26777-9 (PMC8586227; doi:10.1038/s41467-021-26777-9)
Supplement: Supplementary file 1 — Supplementary Information [file 41467_2021_26777_MOESM1_ESM.pdf]

Epigenetic reprogramming of airway macrophages promotes polarization and inflammation in muco-obstructive lung disease

Joschka Hey[1,2,3,11], Michelle Paulsen[3,4,9,11,*], Reka Toth[1,5], Dieter Weichenhan[1], Simone Butz[3,4], Jolanthe Schatterny[3,4], Reinhard Liebers[1,10], Pavlo Lutsik[1], Christoph Plass[1,3,12,*] & Marcus A. Mall[3,4,6,7,8,12,*]

[1]Division of Cancer Epigenomics, German Cancer Research Center (DKFZ), Heidelberg, Germany.

[2]Ruprecht Karl University of Heidelberg, Heidelberg, Germany.

[3]Translational Lung Research Center Heidelberg (TLRC), German Center for Lung Research (DZL), Heidelberg, Germany.

[4]Department of Translational Pulmonology, University of Heidelberg, Heidelberg, Germany.

[5]Division of Molecular Thoracic Oncology, German Cancer Research Center (DKFZ), Heidelberg Germany.

[6]Department of Pediatric Respiratory Medicine, Immunology and Critical Care Medicine, Charité-Universitätsmedizin Berlin, corporate member of Freie Universität Berlin and Humboldt-Universität zu Berlin, Berlin, Germany.

[7]Berlin Institute of Health at Charité – Universitätsmedizin Berlin, Berlin, Germany.

[8]German Center for Lung Research (DZL), associated partner, Berlin, Germany.

[9]Current address: Novo Nordisk Foundation Center for Stem Cell Biology, University of Copenhagen, Copenhagen, Denmark

[10] Current address: National Center for Tumor Diseases (NCT) Heidelberg, Germany

[11]These authors contributed equally.

[12] These authors jointly supervised this work.

[*] Corresponding authors (michelle.paulsen@sund.ku.dk; marcus.mall@charite.de; c.plass@dkfz-heidelberg.de).

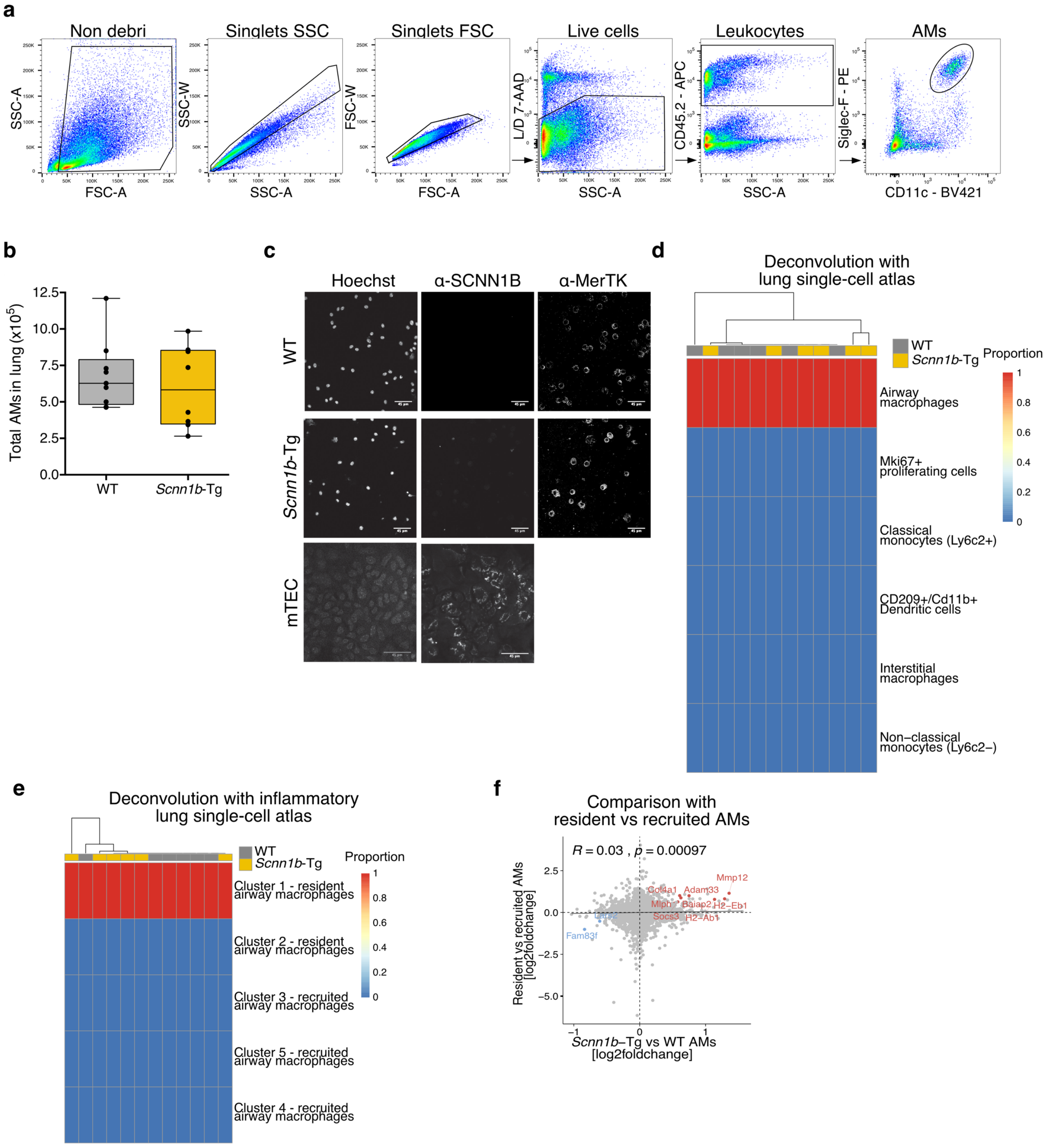

Supplementary Fig. 1

Supplementary Figure 1. The Tissue-resident AM pool is not affected by SCNN1B overexpression. (a) Gating strategy for sorting of airway macrophages (AM) from lungs of *Scnn1b*-transgenic (Tg) vs wild-type (WT) mice. (**b**) Quantification of total AMs in the lung. **(c)** Representative images of n =3 experiments of SCNN1B and MerTK expression on AMs and mouse tracheal epithelial cells (mTEC), by

immunofluorescence microscopy. Deconvolution of *Scnn1b*-Tg and WT AM bulk transcriptomes with (**d**) single-cell RNA sequencing (scRNAseq) of macrophage and monocyte populations, selected from the single-cell atlas of the aging lung and (**e**) scRNAseq of AM clusters, identified in homeostatic and inflammatory mouse lungs, reflecting the cellular origin of AMs. (**f**) Comparison of gene expression changes in *Scnn1b*-Tg vs WT AMs with resident vs recruited AMs. Red dots: significant upregulation in both datasets; blue dots: significant downregulation in both datasets. The gray diagonal represents the linear regression. Shaded areas are the confidence intervals of the correlation coefficient at 95%. Correlation coefficients and *P* values were calculated by the Pearson correlation method. RNA sequencing (RNAseq), n =6 per group. Box plots the largest value within the 1.5 times interquartile range above 75th percentile, 75th percentile, median, 25th percentile, and smallest value within the 1.5 times interquartile range below 25th percentile of **(b)** n=9 WT and n=8 *Scnn1b*-Tg mice.

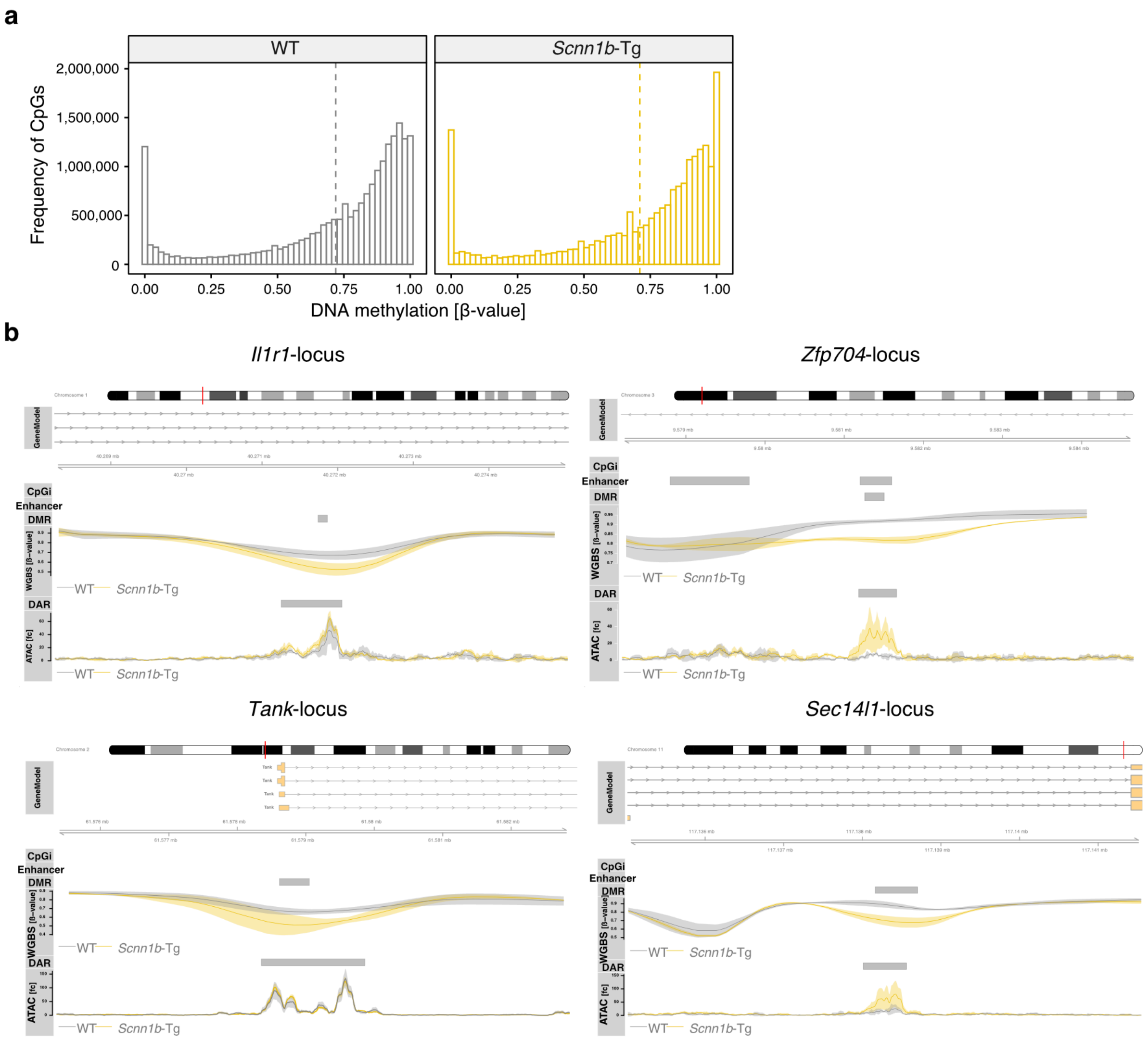

Supplementary Fig. 2

Supplementary Figure 2. AMs from mice with muco-obstructive lung disease are epigenetically distinct from WT AMs. (a) Histogram of the β-value distribution and mean methylation levels in *Scnn1b*-transgenic (Tg) and wild-type (WT) airway macrophages (AM). **(b)** Locus plot of selected differentially methylated regions (DMR) and differentially accessible regions (DAR), visualizing average methylation and chromatin accessibility in *Scnn1b*-Tg AMs and WT AMs. Shaded areas indicate 95% confidence intervals. Tagmentation-based whole-genome bisulfite sequencing (tWGBS), *Scnn1b*-Tg (n =3) vs WT (n =4); Assay for transposase-accessible chromatin sequencing (ATACseq), *Scnn1b*-Tg (n =4) vs WT (n =3).

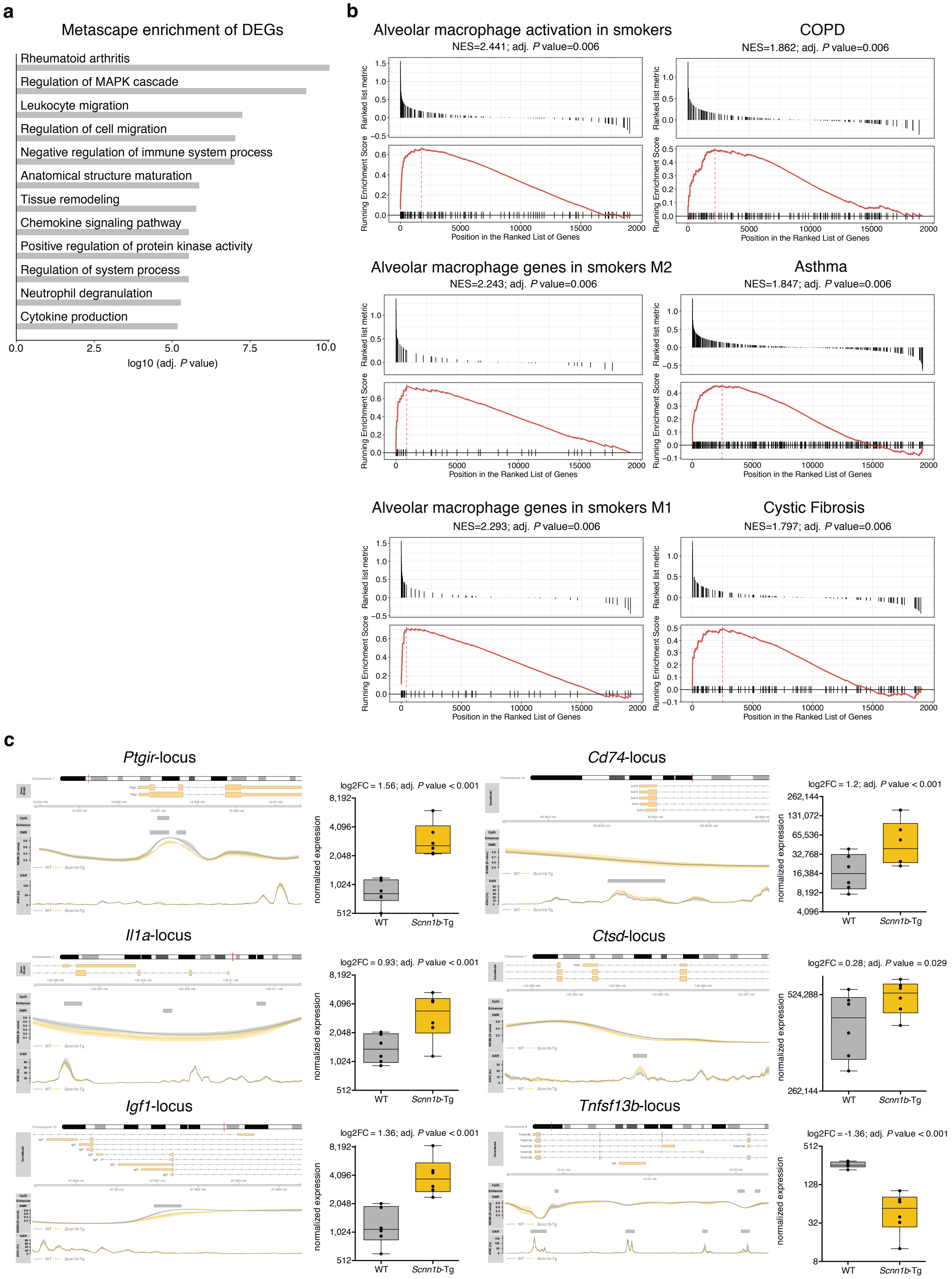

Supplementary Fig. 3

Supplementary Figure 3. Transcriptional activation of *Scnn1b*-Tg AMs coincides with epigenetic patterns of reduced methylation and increased chromatin accessibility. (a) Metascape overrepresentation analysis of DEGs (adjusted (adj.) *P* value <0.1, absolute log2 fold change >0.5). **(b)** Barcode plots of significantly enriched gene sets in *Scnn1b*-transgenic (Tg) airway macrophages (AM). NES: normalized enrichment score. **(c)** Locus plot of selected differentially methylated regions (DMR) and differentially accessible regions (DAR) showing average methylation and chromatin accessibility of *Scnn1b*-Tg and wild-type (WT) AMs. Shaded areas indicate 95% confidence intervals. Normalized gene expression counts for the respective genes are plotted alongside. Adj. *P* values and log2 fold changes (log2FC) were determined by DESeq2. Box plots indicate the largest value within the 1.5 times interquartile range above 75th percentile, 75th percentile, median, 25th percentile, and smallest value within the 1.5 times interquartile range below 25th percentile. RNA sequencing (RNAseq), n =6 per group.

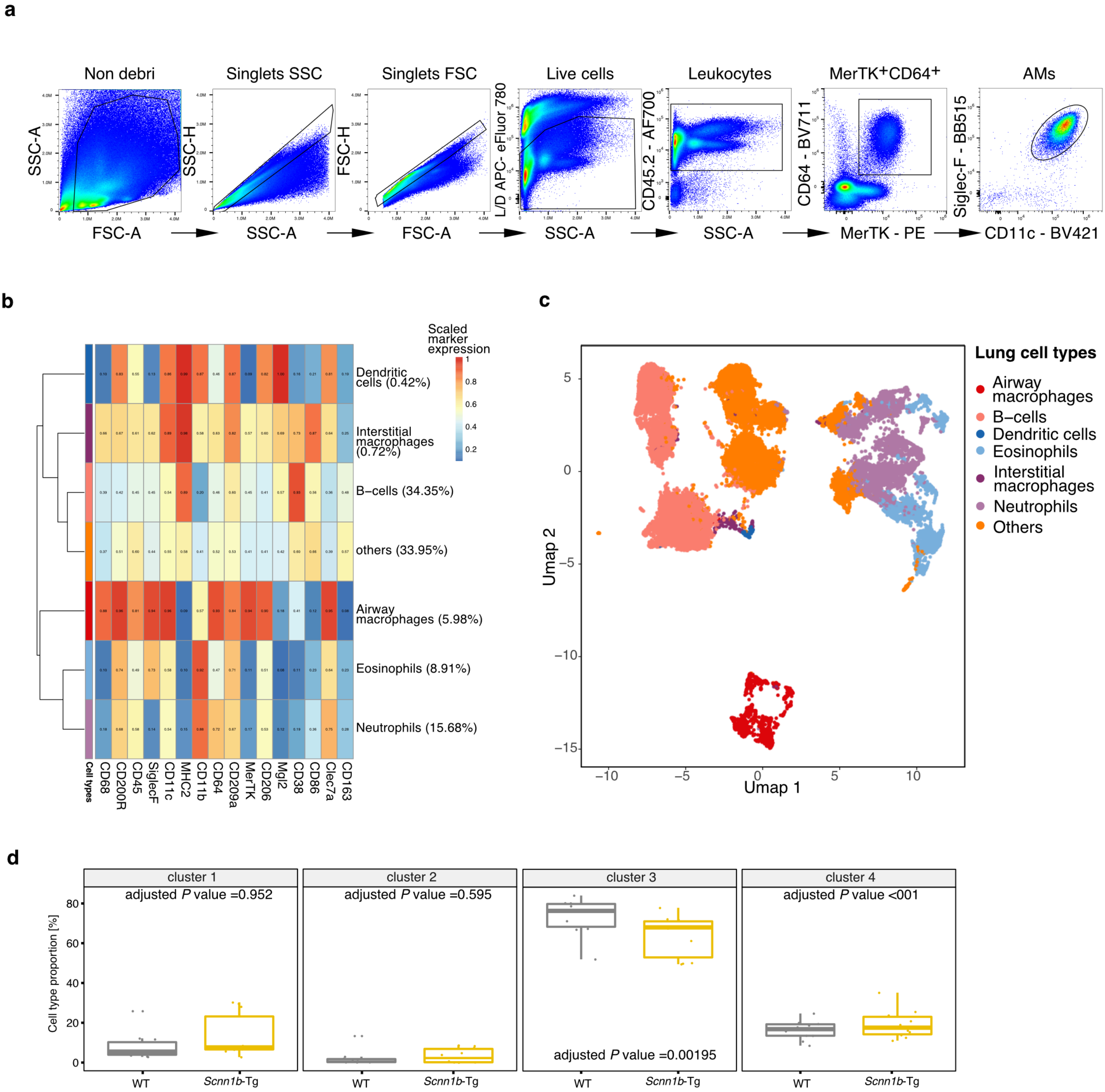

Supplementary Fig. 4

Supplementary Figure 4. Single-cell analysis of macrophage surface marker expression validates enhanced activation of *Scnn1b*-Tg AMs. (a) Gating strategy of *Scnn1b*-transgenic (Tg) and wild-type (WT) lungs to identify airway macrophages (AMs) by high-dimensional flow cytometry. **(b)** Scaled surface marker expression of lung cell types identified via cluster analysis. Average expression over all samples was calculated and applied to hierarchical clustering. **(c)** Uniform Manifold Approximation and Projection (UMAP) of 50,000 randomly sampled *Scnn1b*-Tg and WT leukocytes.

(d) Differential AM cluster abundance. *P* values were generated by fitting a linear model. Box plots indicate the largest value within the 1.5 times interquartile range above 75th percentile, 75th percentile, median, 25th percentile, and smallest value within the 1.5 times interquartile range below 25th percentile. n =10 per group.

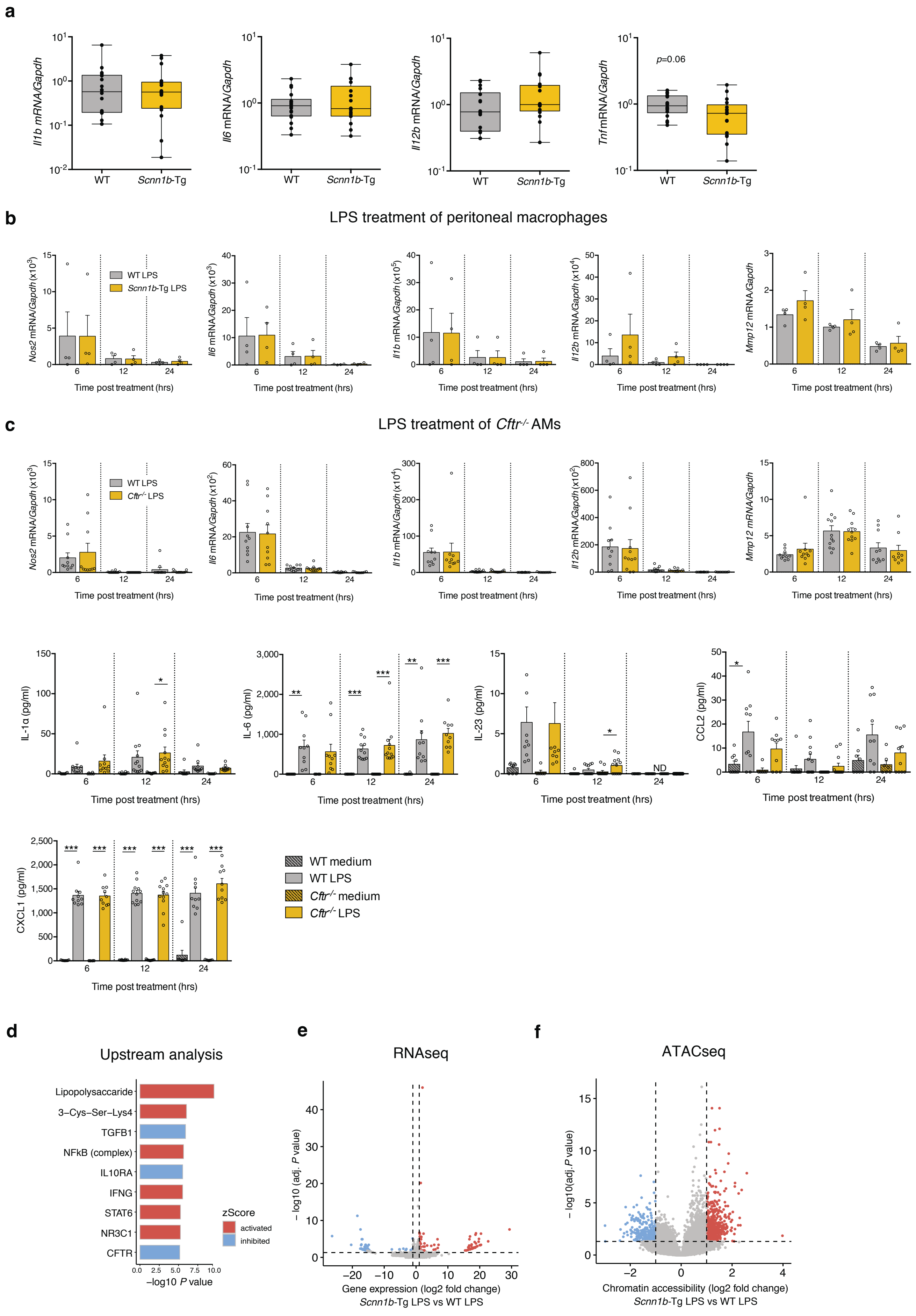

Supplementary Fig. 5

Supplementary Figure 5. AM-specific functions are impaired in *Scnn1b*-Tg mice. **(a)** Baseline gene expression for *Il1b, Il6, Il12b*, and *Tnf* in airway macrophages (AM) from *Scnn1b*-transgenic (Tg) AMs and wild-type (WT) mice. **(b)** Baseline gene expression in peritoneal macrophages from *Scnn1b*-Tg AMs and WT mice, upon treatment with 100 ng/ml lipopolysaccharide (LPS) for 6, 12, and 24 hrs. **(c)** Gene and protein expression of *Cftr*$^{-/-}$ AMs and WT AMs, upon treatment with 100 ng/ml LPS for 6, 12, and 24 hours (hrs). **(d)** Predicted upstream regulator analysis of gene expression changes in *Scnn1b*-Tg and WT AMs, treated for 12 hrs with LPS. **(e)** Volcano plot visualizing the LPS response in AMs (*Scnn1b*-Tg vs WT) on gene expression level. Differentially expressed genes (DEG): adjusted (adj.) *P* value <0.05; log2 fold change >1. Red dots: significantly upregulated genes in *Scnn1b*-Tg AMs; blue dots: significantly upregulated genes in WT AMs. **(f)** Volcano plot visualizing the LPS responses in AMs (*Scnn1b*-Tg vs WT) on chromatin accessibility level. DAR: adj. *P* value <0.05; absolute log2 fold change >1. Red dots: increased accessibility in *Scnn1b*-Tg AMs treated with LPS; blue dots increased accessibility in WT AMs treated with LPS. Box plots indicate the largest value within the 1.5 times interquartile range above 75th percentile, 75th percentile, median, 25th percentile, and smallest value within the 1.5 times interquartile range below 25th percentile. **(a)** n =18 WT, n=17 *Scnn1b*-Tg mice. Bar plots show mean ± SEM of **(b)** n =4 per group and **(c)** gene expression: 6 hrs: n=10 per group; 12 hrs: n=12 WT, n=11 CFTR$^{-/-}$; 24hrs: n=10 WT, n=9 CFTR$^{-/-}$. Protein expression: 6 hrs (med): n=9 WT, n=6 CFTR$^{-/-}$, 6 hrs (LPS): n=10 per group; 12 hrs (med): n=10 WT, n=11 CFTR$^{-/-}$, 12 hrs (LPS): n=12 per group; 24 hrs (med): n=8 WT, n=7 CFTR$^{-/-}$, 24 hrs (LPS): n=10 per group. * *P* value <0.05; ** *P* value <0.01; *** *P* value <0.001 by One-Way ANOVA followed by Tukey´s post hoc test. **(d-f)** n=3 per group. ND, not detectable.

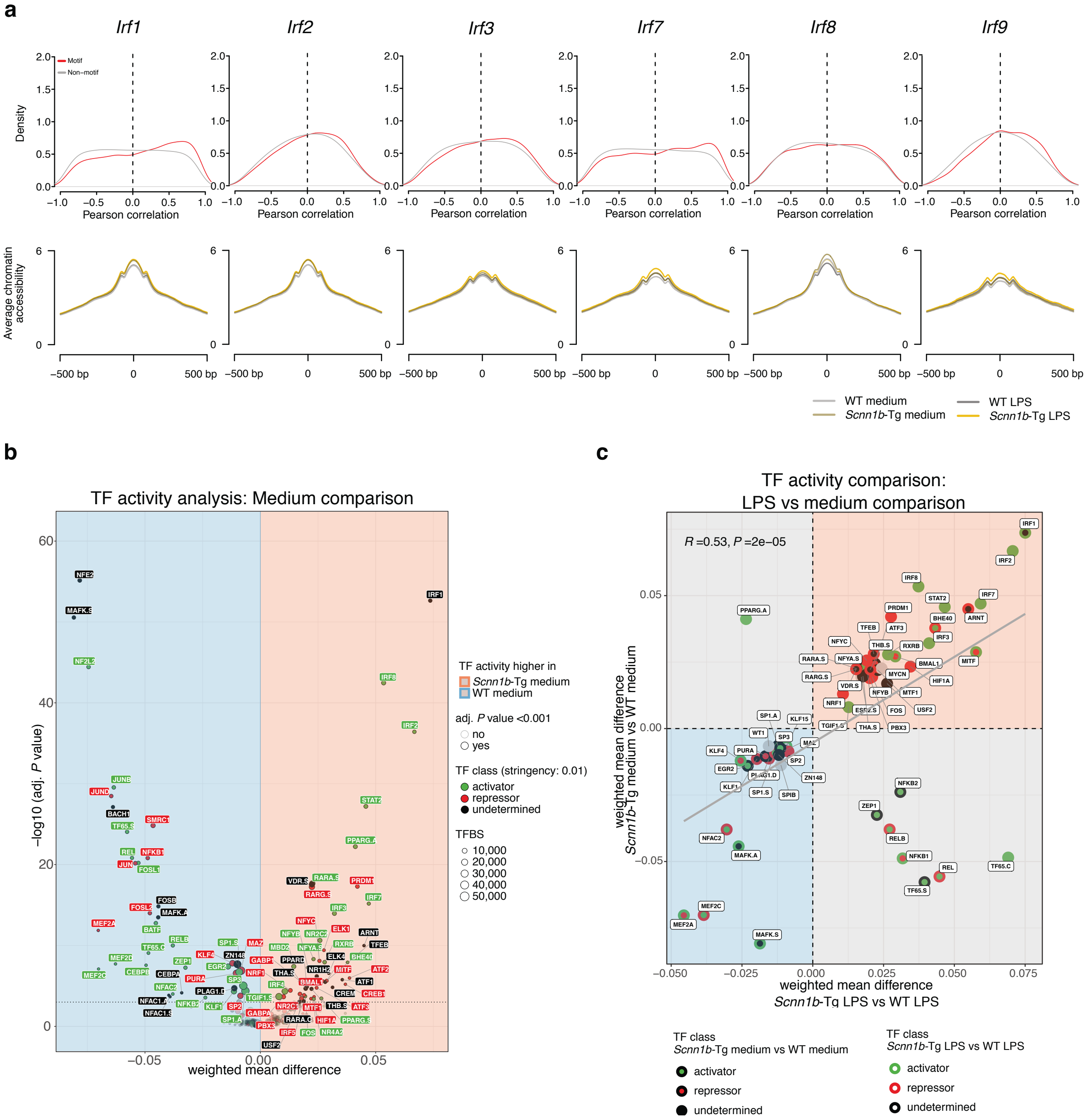

Supplementary Fig. 6

Supplementary Figure 6. Differential transcription factor activity orchestrates LPS responses in *Scnn1b*-Tg AMs. (a) Top panel: Foreground (red) and background (grey) distributions of Pearson correlations between gene expression (n =3) and chromatin accessibility (n =3 per group) at assay for transposase-accessible chromatin sequencing (ATACseq) peaks of differentially active IRF transcription factors (TFs) in *Scnn1b*-transgenic (Tg) vs wild-type (WT) airway macrophages (AM) after 12 hours (hrs) LPS treatment. Lower panel: Profile plot of chromatin accessibility at assay for transposase-accessible chromatin sequencing (ATACseq) peaks with IRF motif. **(b)** Differential transcription factor (TF) activity analysis of medium treated *Scnn1b*-Tg AMs and WT AMs. A positive weighted mean difference indicates increased TF activity in

Scnn1b-Tg AMs cultured in medium for 12 hrs. Size indicates the number of TF binding sites (TFBS). Green labeled TFs are predicted activators, red labeled TFs are predicted repressors, and black labeled TFs have no direction assigned. **(c)** Pearson correlation of significantly enriched TFs (adj. *P* value <0.05) of the LPS and medium comparison of *Scnn1b*-Tg AMs and WT AMs. The gray diagonal represents the linear regression. Correlation coefficients and *P* values were calculated by the Pearson correlation method.

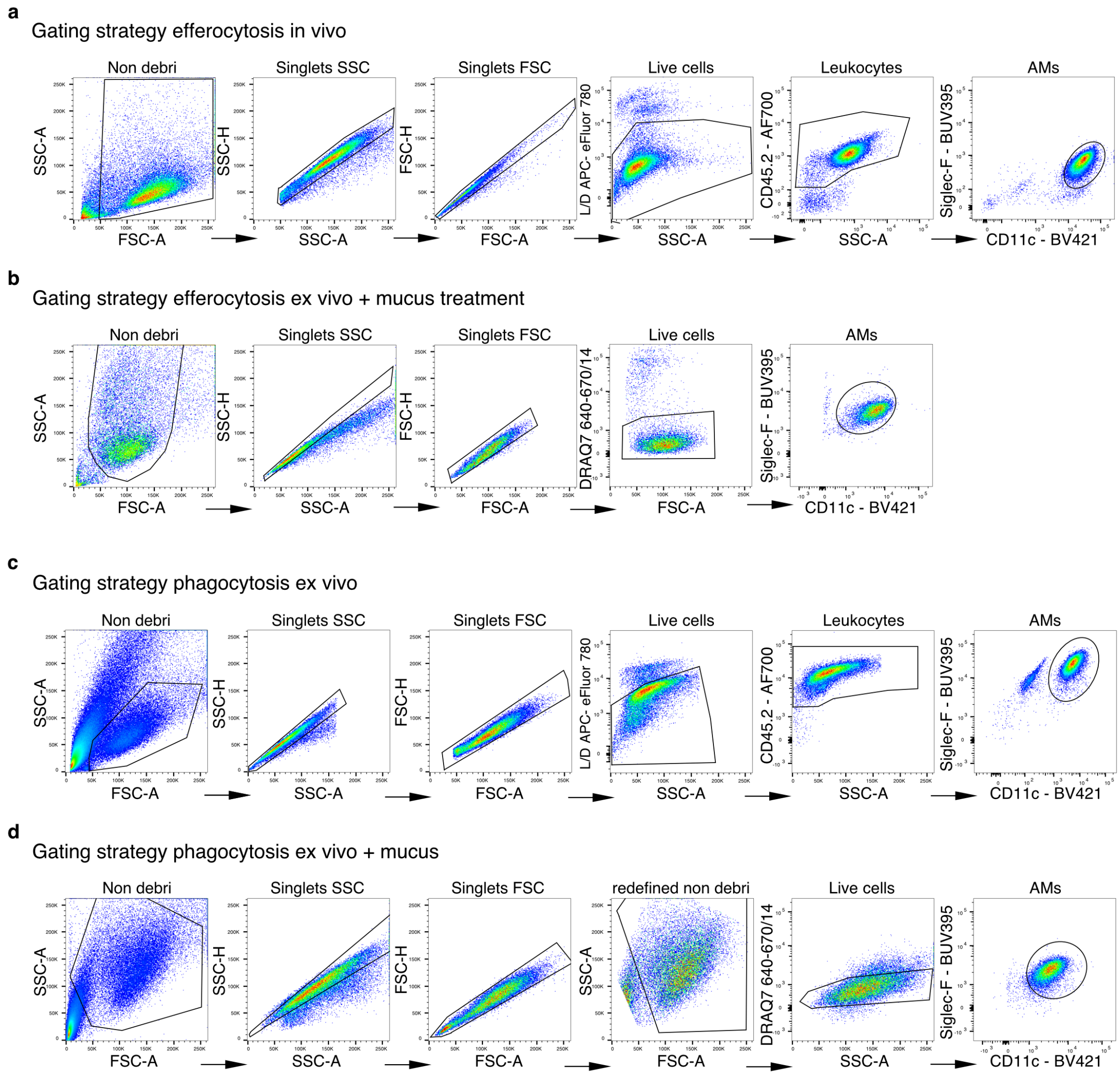

Supplementary Fig. 7

Supplementary Figure 7. Gating strategy for efferocytosis and phagocytosis experiments. (a) Gating strategy for efferocytosis assessment in vivo, using pHRodo Red labeled apoptotic cells. **(b)** Gating strategy for efferocytosis assessment in vitro. upon mucus treatment. **(c)** Gating strategy for phagocytosis assessment, using pHRodo-Red labelled *E. coli* particles. **(d)** Gating strategy for phagocytosis assessment upon mucus treatment.

Supplementary Table 1. List of antibodies for efferocytosis and phagocytosis assay, surface stain, FACS, and Immunocytochemistry.

**Efferocytosis and Phagocytosis**				
**Antibody**	**Clone**	**Fluorophor**	**Dilution for BAL cells**	**Supplier**
Siglec-F	E50-2440	BUV395	1:400	BD Biosciences
CD11c	N418	BV421	1:400	BD Biosciences
CD45.2	104	AF700	1:400	BD Biosciences
CD16/CD32	2.4G2		1:200	BD Biosciences
**Surface stain**				
**Antibody**	**Clone**	**Fluorophor**	**Concentration 1x10^6 lung cells**	**Supplier**
CD206	C068C2	PE-Cy7	1 ug/ml	Biolegend
CD301b (MGL2)	URA-1	PE-Dazzle 594	2 ug/ml	Biolegend
CD369 (CLEC7A)	bg1fpj	PerCp-eFluor710	1 ug/ml	eBiosciences
CD64	X54-5/7.1	BV711	1 ug/ml	Biolegend
MerTK	2B10C42	PE	1 ug/ml	Biolegend
MHCII	M5/114.15.2	BV510	0.25 ug/ml	Biolegend
CD200R	OX110	AF647	2 ug/ml	BD Biosciences
CD38	90	Pacific Blue	1 ug/ml	Biolegend
CD86	GL-1	PerCp-Cy5.5	2 ug/ml	Biolegend
CD68	FA-11	APC	1 ug/ml	Biolegend
CD163	TNKUPJ	Super Bright 436	2 ug/ml	eBiosciences
CD209a	5H10	BV786	2 ug/ml	BD Biosciences
CD11b	M1/70	BV605	0.25 ug/ml	Biolegend
CD11c	N418	BV421	0.5 ug/ml	BD Biosciences
CD45.2	104	AF700	0.5 ug/ml	BD Biosciences
Siglec-F	E50-2440	BB515	0.25 ug/ml	BD Biosciences
CD16/CD32	2.4G2		2.5 ug/ml	BD Biosciences
**FACS**				
**Antibody**	**Clone**	**Fluorophor**	**Concentration 1x10^6 lung cells**	**Supplier**
CD11c	N418	BV421	0.5 ug/ml	BD Biosciences
Siglec-F	E50-2440	PE	0.25 ug/ml	BD Biosciences
CD45.2	104	AF700, APC, PE-Cy7	0.5 ug/ml	BD Biosciences
CD16/CD32	2.4G2		2.5 ug/ml	BD Biosciences
**Immunocytochemistry**				
**Antibody**	**Clone**	**Fluorophor**	**Dilution**	**Supplier**
F(ab´)2 fragment goat anti-rabbit IgG (H+L)	Polyclonal	AF647	1:200	Life Technologies
F(ab´)2 fragment goat anti-rat IgG (H+L)	Polyclonal	AF488	1:300	Life Technologies
F(ab´)2 fragment goat anti-mouse IgG (H+L	Polyclonal	AF488	1:200	Life Technologies
Rat anti-mouse-MerTK	MAB591		1:20	R&D Systems Inc
Mouse anti-mouse-acetylated-a-tubulin	6-11B-1		1:200	Life Technologies
Rabbit anti-mouse-SCNN1B			1:20	provided by Prof. Dr. C. Korbmacher, University Erlangen-Nuremberg

Supplementary Table 2. List of gene expression assays.

**Primer and Probe sets**				
**Gene**	**Primer**	**Probe**	**Supplier**	**Assay ID**
Il6	fwd, 5´-gaggataccactcccaacagacc-3'	5´-FAM-cagaattgccattgcacaa-TAMRA-3´	Eurofins Genomics GmbH	
	rev, 5´-aagtgcatcatcgttgttcataca-3´		Eurofins Genomics GmbH	
Tnf	fwd, 5´-catcttctcaaaattcgagtgacaa-3´	5´-FAM-cacgtcgtagcaaac-TAMRA-3´	Eurofins Genomics GmbH	
	rev, 5´-tgggagtagacaaggtacaaccc-3´		Eurofins Genomics GmbH	
Nos2	Primer sequences are not diclosed by the supplier		Thermo Fisher Scientific	Mm00440502_m1
Il1b			Thermo Fisher Scientific	Mm00434228_m1
Il12			Thermo Fisher Scientific	Mm01288989_m1
Mmp12			Thermo Fisher Scientific	Mm00500554_m1
Arg1			Thermo Fisher Scientific	Mm00475988_m1
Ccl22			Thermo Fisher Scientific	Mm00436439_m1
Ccl17			Thermo Fisher Scientific	Mm01244826_g1
Cd86			Thermo Fisher Scientific	Mm00444543_m1
Cxcr1			Thermo Fisher Scientific	Mm00731329_s1
Trem2			Thermo Fisher Scientific	Mm04209424_g1
Ptgs1			Thermo Fisher Scientific	Mm00477214_m1
Ptgir			Thermo Fisher Scientific	Mm00801939_m1
Anpep			Thermo Fisher Scientific	Mm00476227_m1
Igf1			Thermo Fisher Scientific	Mm00439560_m1
Igf2bp3			Thermo Fisher Scientific	Mm00502738_m1
Gapdh (primer limited)			Thermo Fisher Scientific	4352339E
