# Supplementary material for: Epigenetic reprogramming of airway macrophages promotes polarization and inflammation in muco-obstructive lung disease
Source: Nat Commun. 2021 Nov 11;12:6520. doi: 10.1038/s41467-021-26777-9 (PMC8586227; doi:10.1038/s41467-021-26777-9)
Supplement: Supplementary file 2 — Description of Additional Supplementary Files [file 41467_2021_26777_MOESM2_ESM.pdf]

Description of Additional Supplementary Files

File Name: Supplementary Data 1

Description: List of differentially methylated regions comparing *Scnn1b* transgenic and wild-type airway macrophages at baseline. Differentially methylated regions (DMRs) were defined by DSS. DMRs are characterized by at least three CpGs with adjusted (adj) *P* value <0.05, width of >50 bp, and an average change of methylation >0.1.

File Name: Supplementary Data 2

Description: List of differentially accessible regions comparing *Scnn1b* transgenic and wild-type airway macrophages at baseline. Differentially accessible regions (DARs) were defined by DiffBind. DARs are characterized by an adjusted *P* value <0.05 and log2 fold change >1.

File Name: Supplementary Data 3

Description: List of differentially expressed genes comparing *Scnn1b*-transgenic and wild-type airway macrophages at baseline. Differentially expressed genes (DEGs) were defined by DESeq2. DEGs are characterized by an adjusted *P* value <0.1 and log2 fold change >0.5.

File Name: Supplementary Data 4

Description: List of differentially expressed genes comparing *Scnn1b* transgenic and wild-type airway macrophages post lipopolysaccharide and medium treatment. Differentially expressed genes (DEGs) were defined by DESeq2. DEGs are characterized by an adjusted *P* value <0.05 and log2 fold change >1.

File Name: Supplementary Data 5

Description: List of differentially accessible regions comparing Scnn1b-transgenic and wild-type airway macrophages post lipopolysaccharide and medium treatment. Differentially accessible regions (DARs) were defined by DiffBind. DARs are characterized by an adjusted *P* value <0.05 and log2 fold change >1.

File Name: Supplementary Data 6

Description: HOMER transcription factor motif enrichment results of differentially accessible regions with increased accessibility in *Scnn1b*-transgenic vs wild-type airway macrophages post lipopolysaccharide treatment.

File Name: Supplementary Data 7

Description: List of differentially accessible regions comparing lipopolysaccharide- and medium-treated airway macrophages. Differentially accessible regions (DARs) were defined by DiffBind. DARs are characterized by an adjusted *P* value <0.05 and log2 fold change >2.
