# Supplementary material for: Epigenetic reprogramming of airway macrophages promotes polarization and inflammation in muco-obstructive lung disease
Source: Nat Commun. 2021 Nov 11;12:6520. doi: 10.1038/s41467-021-26777-9 (PMC8586227; doi:10.1038/s41467-021-26777-9)
Supplement: Supplementary file 10 — Reporting Summary [file 41467_2021_26777_MOESM10_ESM.pdf]

nature research

Corresponding author(s): Prof Christoph Plass, Prof Marcus Mall, Dr Michelle Paulsen

Last updated by author(s): Oct 9, 2021

Reporting Summary

Nature Research wishes to improve the reproducibility of the work that we publish. This form provides structure for consistency and transparency in reporting. For further information on Nature Research policies, see our Editorial Policies and the Editorial Policy Checklist.

Statistics

For all statistical analyses, confirm that the following items are present in the figure legend, table legend, main text, or Methods section.

n/a	Confirmed	
☐	☒	The exact sample size (n) for each experimental group/condition, given as a discrete number and unit of measurement
☐	☒	A statement on whether measurements were taken from distinct samples or whether the same sample was measured repeatedly
☐	☒	The statistical test(s) used AND whether they are one- or two-sided<br>*Only common tests should be described solely by name; describe more complex techniques in the Methods section.*
☐	☒	A description of all covariates tested
☐	☒	A description of any assumptions or corrections, such as tests of normality and adjustment for multiple comparisons
☐	☒	A full description of the statistical parameters including central tendency (e.g. means) or other basic estimates (e.g. regression coefficient) AND variation (e.g. standard deviation) or associated estimates of uncertainty (e.g. confidence intervals)
☐	☒	For null hypothesis testing, the test statistic (e.g. F, t, r) with confidence intervals, effect sizes, degrees of freedom and P value noted<br>*Give P values as exact values whenever suitable.*
☒	☐	For Bayesian analysis, information on the choice of priors and Markov chain Monte Carlo settings
☒	☐	For hierarchical and complex designs, identification of the appropriate level for tests and full reporting of outcomes
☐	☒	Estimates of effect sizes (e.g. Cohen's d, Pearson's r), indicating how they were calculated

Our web collection on statistics for biologists contains articles on many of the points above.

Software and code

Policy information about availability of computer code

Data collection	Immunofluorescence pictures were acquired with a Leica TCS SP8 (Leica Microsystems).<br><br>Cells were sorted using a standard BD Fusion equipped with 100-mW 405-nm, 100-mW 488-nm, 80-mW 561-nm, 80-mW 640-nm lasers and an ND2.0 filter in front of the FSC photodiode, a nozzle size of 100 µm, and corresponding BD FACSFlow sheath pressure of 20 psi, matched with a transducer frequency of 32 kHz.<br><br>tWGBS libraries were prepared as previously described (see Methods section) and sequenced paired-end, 125 bp, on one lane of a HiSeq2000 v4 sequencer (Illumina) per sample.<br><br>ATAC libraries were prepared as previously described (see Methods section) and sequenced paired-end, 125 bp, on one lane of a HiSeq2000 v4 sequencer (Illumina).<br><br>RNAseq libraries were prepared with the SMART-Seqv4 Ultra low Input RNA Kit as well as the NEBNext Ultra II Directional RNA Library Prep Kit from Illumina. Sequencing was performed on a High Seq 2000 v4, paired-end, 125 bp platform (Illumina) and Next Seq 500, single-end, 75 bp platform (Illumina), respectively.<br><br>qPCR data were aquired using 7500 Real Time PCR System SDS Software (Applied Biosystems).<br>Cytometric bead array (BD Biosciences) was measured on a BD FortessaLSR.<br><br>Cytokine data were aquired on a Luminex 200.
Data analysis	Immunofluorescence pictures were analyzed using the LASX v3.5.19976 software and FIJI v2.0.0.

Flow cytometry data were analysed using FACSDiva software v8.0, Cytek Spectroflow and FlowJo 10.7 (BD Biosciences).
Surface marker expression data was analysed with FlowSom v1.18.0 and ConsensusClusterPlus v1.5.0.
UMAP was performed by subsampling 5000 cells per samples and the R package umap v0.2.4.1.

Cytometric bead array was quantified according to a standard curve using the BD Cytometric Bead Array FCAP Array Software v3 (BD Biosciences).

Cytokine concentrations were analysed using Bio-Plex Manager 6.2 software.

Sequencing data processing and analytical steps were performed using established software tools.

Processing pipelines:
WGBS processing pipeline: CWL workflow (https://github.com/CompEpigen/WGBS_workflows; Trimmomatic v0.3, bwa mem v0.7.8 , Picard v1.125, MethylDackel v0.3.0)
ATACseq processing pipeline: kundajelab/atac_dnase_pipelines v0.3.0
RNAseq processing pipeline: nf-core RNAseq pipeline v1.2
List of further software:
R v3.6, diffTF v 1.3.3, Graph Pad Prism 6,Ingenuity Pathway Analysis, Metascape (webtool),

List of R libraries:
bsseq v1.20.0, DSS v2.32.0, DiffBind v2.14.0, ChIPseeker v1.22.1, TxDb.Mmusculus.UCSC.mm10.knownGene v3.10.0, DESeq2 v1.26.0, limma v3.42.2, Homer v4.10, Gviz v1.30.3, clusterProfiler v3.12.0, peakSeason v0.1.0, MuSiC v0.1.1, FlowSom v1.18.0, ConsensusClusterPlus v1.5.0.0, umap v0.2.4.1, LOLA

For manuscripts utilizing custom algorithms or software that are central to the research but not yet described in published literature, software must be made available to editors and reviewers. We strongly encourage code deposition in a community repository (e.g. GitHub). See the Nature Research guidelines for submitting code & software for further information.

Data

Policy information about availability of data

All manuscripts must include a data availability statement. This statement should provide the following information, where applicable:
- Accession codes, unique identifiers, or web links for publicly available datasets
- A list of figures that have associated raw data
- A description of any restrictions on data availability

All sequencing data generated in this study has been deposited in the NCBI Gene Expression Omnibus (GEO) under the accession number GSE154808:
WGBS of baseline replicates GSE154803; ATACseq of baseline replicates GSE154804; RNAseq of baseline replicates GSE154805; RNAseq of LPS/medium treated replicates GSE154806; ATACseq of LPS/medium treated replicates GSE154807
The SubSeries include the results of downstream statistical analysis, such as differentially expressed genes, differentially methylated regions as well as differentially accessible regions.

mm10 reference genome was downloaded from ENCODE (https://www.encodeproject.org/files/mm10_no_alt_analysis_set_ENCODE/@@download/mm10_no_alt_analysis_set_ENCODE.fasta.gz).

Gen code gene annotation release M20 was downloaded from Gencode (https://www.gencodegenes.org/mouse/release_M20.html).

Furthermore, we used custom gene sets from Saini et al., 2014.

In silico predicted transcription factor binding sites were aquired from the HOCOMOCO 10 database (https://hocomoco11.autosome.ru/downloads_v10).

To cluster differentially active transcription factors, the clustering results of RSAT were used.

Field-specific reporting

Please select the one below that is the best fit for your research. If you are not sure, read the appropriate sections before making your selection.

☒ Life sciences ☐ Behavioural & social sciences ☐ Ecological, evolutionary & environmental sciences

For a reference copy of the document with all sections, see nature.com/documents/nr-reporting-summary-flat.pdf

Life sciences study design

All studies must disclose on these points even when the disclosure is negative.

Sample size	Sample sizes were determined based on prior knowledge of good sample sizes (Trojanek et al. 2014, Saini et al. 2014, Mall et al. 2004) to ensure adequate data for reliable assessments as well as feasibility sequencing data generation. Sample sizes are indicated in the figure legends.
Data exclusions	Data was excluded based on QC results for individual assays. WGBS samples used for downstream analysis had a bisulfite conversion rate of above 98% and more than 95% of all reference CpGs were covered. Each baseline ATACseq replicate achieved a minimum of 50 million non-duplicated, non-mitochondrial reads. The irreproducible discovery rate was less than two for each Scnn1b-Tg and WT group of replicates. The fraction of reads in called peaks was above 0.5. All RNA samples reached an RNA integrity number (RIN) > 8.5.
Replication	All experiments were performed in at least three biological replicates and specific sample sizes are mentioned in the figure legends. Most experiments contain statistical analysis and significances of the results are indicated in the figure or figure legend. For immunofluorescence data and flow cytometry, representative images are shown.
Randomization	Our samples were derived from two genetically distinct groups of animals. To maintain the colony, wild-type (WT) and Scnn1b-Transgenic (Tg) mice were bred together, resulting in litters comprised of mice with Scnn1b-Tg and WT genetic background. In studies involving baseline characterization of animal groups, we allocated litter mates of different genotype into one experiment, to control for husbandry differences between cages. Sex bias was controlled by allocating equal numbers of male and female mice into the experiment (for sequencing experiments only female mice were used). The same procedure was used to allocate animals in LPS or mucus treatment studies.
Blinding	To distinguish Scnn1b-transgenic (Tg) and wild-type (WT) animals in the same cage, mice were genotyped and labeled by ear marks. The scientist conducting the experiments had to verify the ear mark on the mouse to use the correct genotype. Our studies did not involve behavioral assessment or treatment outcome in live animals, for which unblinded studies can induce a high bias in observers. Upon sample collection all allocated groups were known to the researcher. Downstream molecular analysis and statistical analysis should provide objective outcomes.

Reporting for specific materials, systems and methods

We require information from authors about some types of materials, experimental systems and methods used in many studies. Here, indicate whether each material, system or method listed is relevant to your study. If you are not sure if a list item applies to your research, read the appropriate section before selecting a response.

Materials & experimental systems

n/a	Involved in the study	
☐	☒	Antibodies
☐	☒	Eukaryotic cell lines
☒	☐	Palaeontology and archaeology
☐	☒	Animals and other organisms
☒	☐	Human research participants
☒	☐	Clinical data
☒	☐	Dual use research of concern

Methods

n/a	Involved in the study	
☒	☐	ChIP-seq
☐	☒	Flow cytometry
☒	☐	MRI-based neuroimaging

Antibodies

Antibodies used

Efferocytosis and Phagocytosis:
Siglec-F, clone E50-2440, BUV395, 1:400, supplier BD Biosciences, Cat. Nr. 740280
CD11c, clone N418, BV421, 1:400, supplier BD Biosciences, Cat. Nr. 565452
CD45.2, clone 104, AF700, 1:400, supplier BD Biosciences, Cat. Nr. 560693
CD16/CD32 (Mouse BD Fc Block), clone 2.4G2, 1:200, supplier BD Biosciences, Cat. Nr. 553142
Surface staining:
CD206, clone C068C2, PE-Cy7, 1 ug/ml, supplier Biolegend, Cat. Nr. 141720
CD301b (MGL2), clone URA-1, PE-Dazzle, 594 2 ug/ml, supplier Biolegend, Cat. Nr. 146816
CD369 (CLEC7A) , clone bg1fpj, PerCp-eFluor710, 1 ug/ml, supplier eBiosciences, Cat. Nr. 46-5859-82
CD64, clone X54-5/7.1, BV711, 1 ug/ml, supplier Biolegend, Cat. Nr. 139311
MerTK, clone 2B10C42, PE, 1 ug/ml, supplier Biolegend, Cat. Nr. 151506
MHCII, clone M5/114.15.2, BV510, 0.25 ug/ml, supplier Biolegend, Cat. Nr. 107636
CD200R, clone OX110, AF647, 2 ug/ml, supplier BD Biosciences, Cat. Nr. 566345
CD38, clone 90, Pacific Blue, 1 ug/ml, supplier Biolegend, Cat. Nr. 102720
CD86, clone GL-1, PerCp-Cy5.5, 2 ug/ml, supplier Biolegend, Cat. Nr. 105028
CD68, clone FA-11, APC, 1 ug/ml, supplier Biolegend, Cat. Nr. 137008
CD163, clone TNKUPJ, Super Bright 436, 2 ug/ml, supplier eBiosciences, Cat. Nr. 62-1631-82
CD209a, clone 5H10, BV786, 2 ug/ml, supplier BD Biosciences, Cat. Nr. 741023

CD11b, clone M1/70, BV605, 0.25 ug/ml, supplier Biolegend, Cat. Nr. 101257
CD11c, clone N418, BV421, 0.5 ug/ml, supplier BD Biosciences, Cat. Nr. 565452
CD45.2, clone 104, AF700, 0.5 ug/ml, supplier BD Biosciences, Cat. Nr. 560693
Siglec-F, clone E50-2440, BB515, 0.25 ug/ml, supplier BD Biosciences, Cat. Nr. 564514
CD16/CD32 (Mouse BD Fc Block), clone 2.4G2, 2.5 ug/ml, supplier BD Biosciences, Cat. Nr. 553142
FACS:
CD11c, clone N418, BV421, 0.5 ug/ml, supplier BD Biosciences, Cat. Nr. 565452
Siglec-F, clone E50-2440, PE, 0.25 ug/ml, supplier BD Biosciences, Cat. Nr. 552126
CD45.2, clone 104, AF700, APC, PE-Cy7, 0.5 ug/ml, supplier BD Biosciences, Cat. Nr. 560693 (AF700), 558702 (APC), 560696 (PE-Cy7)
CD16/CD32 (Mouse BD Fc Block), clone 2.4G2, 2.5 ug/ml, supplier BD Biosciences, Cat. Nr. 553142
Fluorescence microscopy:
F(ab´)2 fragment goat anti-rabbit IgG (H+L), polyclonal, AF647, 1:200, supplier Life Technologies, Cat. Nr. A-21246
F(ab´)2 fragment goat anti-rat IgG (H+L), polyclonal, AF488, 1:300, supplier Life Technologies, Cat. Nr. A-11006
F(ab´)2 fragment goat anti-mouse IgG (H+L), polyclonal, AF488, 1:200, supplier Life Technologies, Cat. Nr. A48286
Rat anti-mouse-MerTK, clone MAB591, 1:20, supplier R&D Systems Inc., Cat. Nr. MAB591-100
Mouse anti-mouse-acetylated-a-tubulin, clone 6-11B-1, 1:200, supplier Life Technologies, Cat. Nr. 32-2700
Rabbit anti-mouse-SCNN1B, 1:20

Validation

SCNN1B was kindly provided by Prof. Dr. C. Korbmacher, University Erlangen-Nuremberg and previously used in Seys et al., 2015 (doi:10.1371/journal.pone.0129897).
Siglec-F clone E50-2440 was validated by the supplier on bone-marrow myeloid cells and cited in the following publication: Angata T, et al. J Biol Chem. doi: 10.1074/jbc.M108573200.
CD11c clone N418 was validated by the supplier on C57BL/6 mouse splenic leucocyte subsets and cited in the following publications: Crowley MT, et al. J Immunol Methods. doi: 10.1016/0022-1759(90)90318-p; Metlay JP, et al. J Exp Med.; doi: 10.1084/jem.171.5.1753.
CD45.2 clone 104 was validated by the supplier on splenocytes from BALB/c and SJL mice and cited in the following publications: Greimers R, et al. Cytometry. doi: 10.1002/(SICI)1097-0320(19960301)23:3<205::AID-CYTO4>3.0.CO;2-H; Johnson P, et al. J Exp Med. doi: 10.1084/jem.169.3.1179.
CD16/32 clone 2.4G2 was validated by the supplier on mouse spleen cells and cited in the following publication: Araujo-Jorge T, et al. Infect Immun. doi: 10.1128/iai.61.11.4925-4928.1993.
CD206 clone C068C2 was validated by the supplier on BALB/c peritoneal macrophages and cited in the following publications: Xiang W, et al. Nat Commun. doi: 10.1038/s41467-018-04999-8; Gubin MM, et al. Cell. doi: 10.1016/j.cell.2018.09.030.
CD301b (MGL2) clone URA-1 was validated by the supplier on C57BL/6 bone marrow-derived dendritic cells and cited in the following publications: Sil P, et al. J Allergy Clin doi: 10.1016/j.jaci.2019.11.041; Utz SG, et al. Cell. doi: 10.1016/j.cell.2020.03.021.
CD64 clone X54-5/7.1 was validated by the supplier on C57BL/6 mouse bone-marrow cells and cited in the following publications: Buschor S, et al. PLoS Pathogens. doi: 10.1371/journal.ppat.1006476; Gentek R, et al. Immunity. doi: 10.1016/j.immuni.2018.04.025.
MerTK clone 2B10C42 was validated by the supplier on C57BL/6 mouse splenocytes and cited in the following publications: Martínez-López M et al. Immunity. doi: 10.1016/j.immuni.2018.12.020; Hou X, et al. Cell Reports. doi: 10.1016/j.celrep.2019.06.007.
MHCII clone M5/114.15.2 was validated by the supplier on C57BL/6 mouse splenocytes and cited in the following publications: Eddy W, et al. The Journal of Immunology. doi: 10.4049/jimmunol.1601777; Hammer A, et al. Front Immunol. doi: 10.3389/fimmu.2017.01922.
CD38 clone 90 was validated by the supplier on C57BL/6 mouse splenocytes and cited in the following publications: Yang P et al. Cell Syst. doi: 10.1016/j.cels.2019.03.012; Adachi Y, et al. Nat Commun. doi: 10.1038/s41467-019-11821-6.
CD86 clone GL-1 was validated by the supplier on LPS-stimulated C57BL/6 mouse splenocytes and cited in the following publications: Raso F, et al. J Clin Invest. doi: 10.1172/JCI99597; Hammer A, et al. Front Immunol. doi: 10.3389/fimmu.2017.01922
CD68 clone FA-11 was validated by the supplier on Thioglycolate-elicited BALB/c peritoneal macrophages and cited in the following publications: Wang X, et al. 2017. Br J Pharmacol. 10.1111/bph.13786; Wang Y et al. Cell. doi: 10.1016/j.cell.2017.08.041.
CD11b clone M1/70 was validated by the supplier on C57BL/6 mouse bone-marrow cells and cited in the following publications: Cohen M et al. Cell. doi: 10.1016/j.cell.2018.09.009; Campbell C et al. Immunity. doi: 10.1016/j.immuni.2018.04.013.
CD369 (CLEC7A) clone bg1fp was validated by the supplier on BALB/c lysed whole blood.
CD200R clone OX110 was validated by the supplier on mouse bone marrow and cited in the following publications: Jenmalm MC, et al. J Immunol. doi: 10.4049/jimmunol.176.1.191; Wright GJ, et al. J Immunol. doi: 10.4049/jimmunol.171.6.3034.
CD163 clone TNKUPJ was validated by the supplier on BALB/c mouse splenocytes.

CD209a clone 5H10 was cited in the following publications: Baribaud F, et al. J Virol. doi: 10.1128/JVI.75.21.10281-10289.2001.; Caminschi I, et al. Mol Immunol. doi: 10.1016/s0161-5890(01)00067-0.
F(ab´)2 fragment goat anti-rabbit IgG (H+L) was validated by the supplier by immunofluorescence analysis using A-431 cells stained with EGFR Rabbit Monoclonal Primary Antibody and cited in the following publication: Quadrato G, et al. Nature. doi: 10.1038/nature22047
F(ab´)2 fragment goat anti-rat IgG (H+L) was validated by the supplier by immunofluorescence analysis of Polyoma Virus Medium T in PyMT+ mammary tumor cells and mammary gland lymph node and cited in the following publication: Nüchel J, et al. Autophagy. doi: 10.1080/15548627.2017.1422850.
F(ab´)2 fragment goat anti-mouse IgG (H+L) was validated by the supplier by immunofluorescent analysis of tubulin in HeLa cells.
Rat anti-mouse-MerTK clone MAB591 was validated by the supplier in immersion fixed J774A.1 mouse cell line and cited in the following publication: Zhao GJ, et al. Mediators Inflamm. doi: 10.1155/2017/6848430.; Deng T, et al. Immunology. doi: 10.1111/j.1365-2567.2011.03511.x.
Mouse anti-mouse-acetylated-a-tubulin clone 6-11B-1 was validated by the supplier by immunofluorescence analysis on HeLa cells and cited in the following publication: Bosakova M, et al. EMBO Mol Med. doi: 10.15252/emmm.201911739; Munro I, et al. J Microsc. doi: 10.1111/jmi.12772.

Eukaryotic cell lines

Policy information about cell lines

Cell line source(s)	LA-4 (ATCC CCL-196)
Authentication	Cells were freshly purchased from ATCC, expanded for stock generation according to ATCC protocols, and used for experimental procedures. Freshly purchased and expanded cell line was not further authenticated in our laboratory.
Mycoplasma contamination	Freshly purchased and expanded cell line was not further tested.
Commonly misidentified lines (See ICLAC register)	No commonly misidentified cell lines were used.

Animals and other organisms

Policy information about studies involving animals; ARRIVE guidelines recommended for reporting animal research

Laboratory animals	Scnn1b-Tg mice on C57BL/6 background and gut-corrected Cftr-/- mice (Cftrtm1Unc Tg(FABPCFTR) on C57BL/6 background were bred in-house under specific pathogen-free conditions and genotyped as previously described . For experiments involving FACS sorting, 6-week old female mice were used. For all other experiments gender matched 6-week old mice were used. WT littermates were used as control animals. Mice were housed at room temperatures of 22 degree C +-2 degree C with 50-60% humidity and kept on a 12/12 h light/dark cycle with continuous access to food and water.
Wild animals	The study did not involve wild animals
Field-collected samples	The study did not involve samples collected from the field.
Ethics oversight	All animal studies were approved by the animal welfare authorities responsible for the University of Heidelberg (Regierungspräsidium Karlsruhe, Karlsruhe, Germany).

Note that full information on the approval of the study protocol must also be provided in the manuscript.

Flow Cytometry

Plots

Confirm that:

- [x] The axis labels state the marker and fluorochrome used (e.g. CD4-FITC).
- [x] The axis scales are clearly visible. Include numbers along axes only for bottom left plot of group (a 'group' is an analysis of identical markers).
- [x] All plots are contour plots with outliers or pseudocolor plots.
- [x] A numerical value for number of cells or percentage (with statistics) is provided.

Methodology

Sample preparation	For all experiments using flow cytometry and FACS whole lungs were extracted from mice. For sort preparations lungs were digested with Dispase and after red blood cell lysis the single cell suspension was enriched by magnetic bead separation using CD45+ magnetic beads (Miltenyi Biotech). For flow cytometry other then sorts, lungs were digested with Collagenase D to obtain single cell suspensions and red blood cells were lysed.
Instrument	Samples were sorted using a standard BD Fusion equipped with 100-mW 405-nm, 100-mW 488-nm, 80-mW 561-nm, 80-mW 640-nm lasers and an ND2.0 filter in front of the FSC photodiode, a nozzle size of 100 µm, and corresponding BD FACSFlow sheath pressure of 20 psi, matched with a transducer frequency of 32 kHz.<br>For surface staining samples were acquired on a standard 405/488/561/640nm laser engine CYTEK Aurora.<br>For efferocytosis and phagocytosis experiments samples were acquired on a BD LSRFortessa equipped with 20-mW 355-nm, 50-mW 405-nm, 50-mW 488-nm, 50-mW 561-nm, 40-mW 640-nm lasers and an ND1.0 filter in front of the FSC photodiode.
Software	Data were analysed using FACSDiva software v8.0, Cytek Spectroflow and FlowJo 10.7.
Cell population abundance	Sorted AMs represented 9-15 % of leukocyte population. Purity check of sorted cells was performed on selected samples from each run confirming purities ranging 95-99%.
Gating strategy	Gating was performed according to ISAC MIFlowcyt standards. Gating on non-debri cells was resolved on FSC-A and SSC-A. Following doublet exclusion on SSC-H and SSC-A, and FSC-H and FSC-A. The obtained singlet cells were further gated on viability, using 7-AAD or l/d fixable dye eFluor780. The gating strategies following doublet exclusion are shown in supplementary figures. FMO controls were used as gating controls, when low spectral overlap did not skew the populations. This was the case for all chosen colors, and antibodies matches in our experimental setup.

- [x] Tick this box to confirm that a figure exemplifying the gating strategy is provided in the Supplementary Information.
